# Mitochondrial genome diversity on the Central Siberian Plateau with particular reference to the prehistory of northernmost Eurasia

**DOI:** 10.1371/journal.pone.0244228

**Published:** 2021-01-28

**Authors:** Stanislav V. Dryomov, Azhar M. Nazhmidenova, Elena B. Starikovskaya, Sofia A. Shalaurova, Nadin Rohland, Swapan Mallick, Rebecca Bernardos, Anatoly P. Derevianko, David Reich, Rem I. Sukernik

**Affiliations:** 1 Laboratory of Human Molecular Genetics, Institute of Molecular and Cellular Biology, SBRAS, Novosibirsk, Russian Federation; 2 Department of Genetics, Harvard Medical School, Boston, Massachusetts, United States of America; 3 Broad Institute of Harvard and MIT, Cambridge, Massachusetts, United States of America; 4 Howard Hughes Medical Institute, Harvard Medical School, Boston, Massachusetts, United States of America; 5 Institute of Archaeology and Ethnography, SBRAS, Novosibirsk, Russian Federation; University of Florence, ITALY

## Abstract

The Central Siberian Plateau was the last geographic area in Eurasia to become habitable by modern humans after the Last Glacial Maximum (LGM). Through a comprehensive dataset of mitochondrial DNA (mtDNA) genomes retained in the remnats of earlier (“Old”) Siberians, primarily the Ket, Tofalar, and Todzhi, we explored genetic links between the Yenisei-Sayan region and Northeast Eurasia (best represented by the Yukaghir) over the last 10,000 years. We generated 218 new complete mtDNA sequences and placed them into compound phylogenies with 7 newly obtained and 70 published ancient mitochondrial genomes. We have considerably extended the mtDNA sequence diversity (at the entire mtDNA genome level) of autochthonous Siberians, which remain poorly sampled, and these new data may have a broad impact on the study of human migration. We compared present-day mtDNA diversity in these groups with complete mitochondrial genomes from ancient samples from the region and placed the samples into combined genealogical trees. The resulting components were used to clarify the origins and expansion history of mtDNA lineages that evolved in the refugia of south-central Siberia and beyond, as well as multiple phases of connection between this region and distant parts of Eurasia.

## Introduction

Although modern humans began colonizing Subarctic and Arctic Siberia 45 kya, most archeological sites postdate 12 kya, with the exception of episodic incursions to the north of Eurasia during the warm phases, with the settlements primarily limited to cryptic refugia in both eastern Europe and Asia (reviewed by [1]). During the last millennium, the Central Siberian Plateau, bounded by the Yenisei River to the west, the North Siberian Lowland to the north, the Verkhoyansk Mountains to the east, and the Sayan Mountains and Lake Baikal to the south, has been shaped by key episodes that have had major impacts on human migrations, admixture, and population replacement. At the end of the 15^th^ century, when Russians first crossed the Ural Mountains in substantial numbers, dozens of indigenous tribes and bands with markedly different ways of life and subsistence were widespread throughout Siberia [2, 3]. Soon after, some of these groups went extinct or underwent marked decimation and admixture, while others only diminished in number, their territories shrinking to small patches of their former distributions. Two strata are recognized in the aboriginal Siberian population: an ancient layer and a comparatively new one, or the “Old Siberians” versus “Neo-Siberians” [4, 5]. Most early Siberians who spoke languages belonging to linguistic outliers, such as Omok (an extinct Yukaghir language spoken as late as the 18^th^ century in the lower Kolyma and Indigirka valleys), have been greatly reduced in number, and their cultures have been dominated by the more populous Neo-Siberians, i.e., Tungusic, Turkic and Mongolian tribes, all relative newcomers to the region [2–6]. Nonetheless, the Old Siberians still persist in remote “pockets” of Siberia in the form of anthropological isolates, usually representing the now-amalgamating remnants of small, previously distinct tribes and lineages. The study of mitochondrial DNA (mtDNA) variation to ascertain the origin and affinities of these groups, as well as their relationships with the first Americans, has been intensive since the early 1990s [7–11]. Candidate founding lineages for Native American mtDNA haplogroups were determined by identifying similar haplogroups in Eurasia [12–17]. For instance, unique mtDNA sequences (haplogroups B4b1a, D4h2, and C1a) linked to Native American B2, D4h3a, and C1b’c’d, respectively, were identified using complete mtDNA sequences [12–14, 16]. A complete catalog of mtDNA variation in Old Siberians is therefore important not only for understanding the specific history of Siberia but also for understanding the ancestry of peoples of the New World.

**Fig 1:**
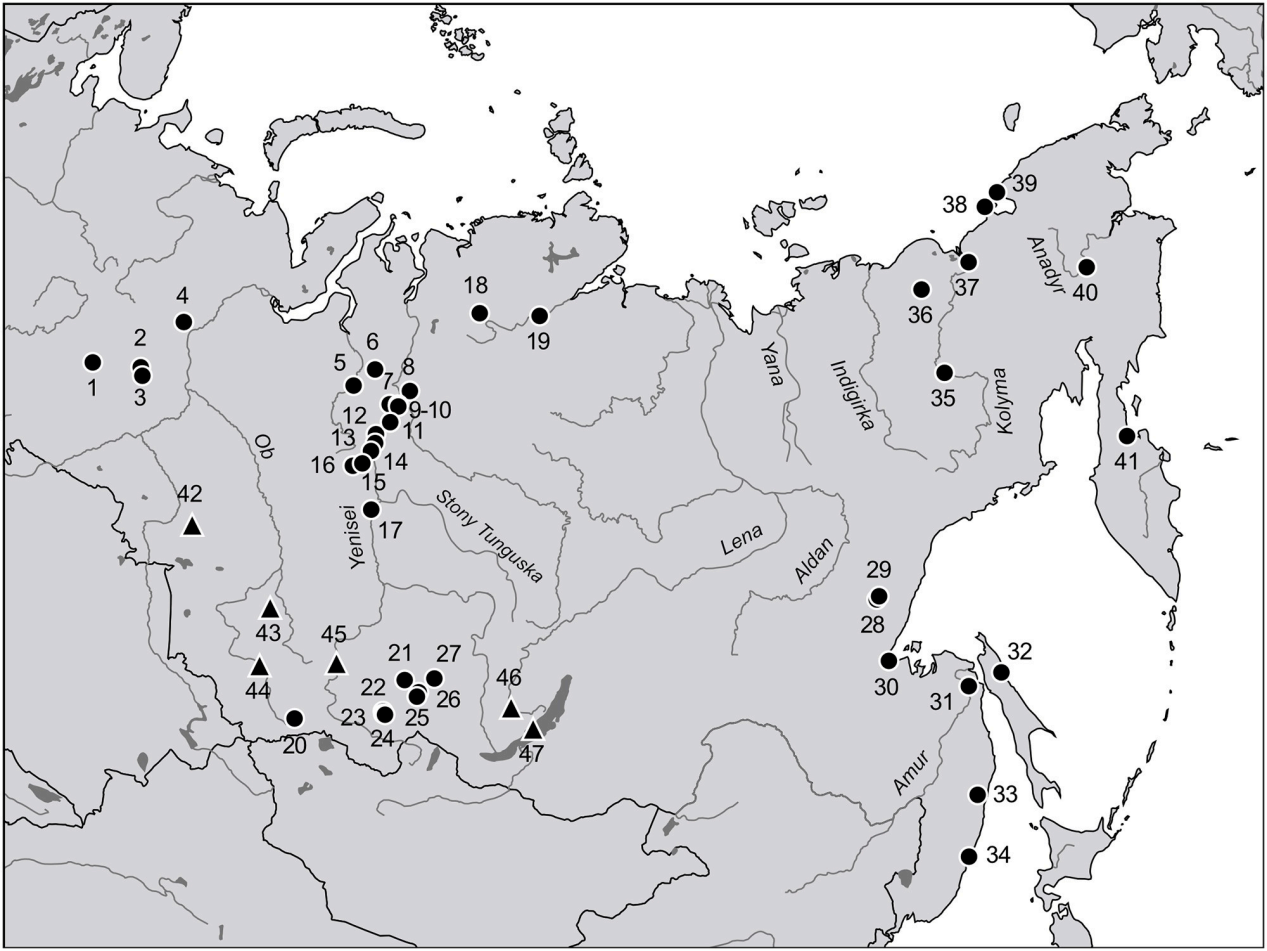
Map of Siberia. Black circles mark the settlements of sampling expeditions listed in S1 Table in S1 File: 1—v. Verkhoturye; 2—v. Shaim; 3—v. Urai; 4—v. Aneyeva; 5—v. Krasnoselkup; 6—v. Sovetskaya Rechka; 7—v. Farkovo; 8—v. Maduika; 9—v. Selivanikha; 10—v. Turukhansk; 11—v. Baikha; 12—Pakulikha river; 13—v. Surgutikha; 14—v. Kangatovo; 15—v. Yelogui; 16—v. Kellog; 17—v. Vorogovo; 18—v. Ust’-Avam; 19—factoriya Novaya; 20—v. Bai-Tal; 21—v. Verkhnyaya Gutara; 22—v. Iiy; 23—v. Toora-Hem; 24—v. Adyr-Kezhig; 25—v. Alygdzher; 26—v. Nerkha; 27—v. Kushun; 28—v. Dzhigda; 29—v. Nelkan; 30—v. Chumikan; 31—v. Bogorodskoye; 32—v. Val; 33—v. Agzu; 34—v. Ternei; 35—v. Nelemnoye; 36—v. Andrushkino; 37—v. Cherskiy; 38—v. Ayon; 39—v. Yanranay; 40—v. Markovo; 41—Karaginskiy district; Black triangles denote locations of the ancient specimens generated through the course of this study and listed in S2 Table in S1 File: 42—Korchugan-1; 43—Vas’kovo-4; 44—Solontsy-5; 45—Tepsei-III; 46—Obkhoy; 47—Khuzhir-2.

A central concern of this study is disentangling ancestries from different sources that mixed at different time points in the past to provide a better understanding of the genetic interactions between and within Siberian populations. Accordingly, we focused on mtDNA genome diversity in Yenisei-Sayan autochthonous populations, primarily the Ket, Tofalar, and Todzhi, as well as the Yukaghir (Fig 1). The Ket are now the sole surviving group of the Yeniseian language family, speaking a unique language that does not easily fit into any known phyla and is unrelated to any other Siberian language [18]. Their related tribes, the Assan, Arin, and Kott, disappeared approximately 200 years after their first contact with Russians [5]. In this region, the Ket are the last tribe to retain their original language and until recent times subsisted entirely on hunting, fishing, and the gathering of wild plants [2, 19, 20]. The Tofalar and Todzhi languages, whose members originally spoke Samoyed, belong to the subgroup of Turkic languages confined to the upper Yenisei area [21–23], where their speakers may have had ample opportunity to exchange genes with the Ket and/or related tribes [5]. Considering current knowledge about the Old Siberians, we see the amalgamated remnants of the Yukaghir confined to an ~200-kilometer-wide strip of land along the Arctic Ocean between the Lena and Kolyma Rivers and several enclaves to the east and south that were surrounded by the Chukchi and Tungus [4, 5, 24, 25]. The Tungus encroachment on the Yukaghir intensified after the 15^th^ century due to the colonization of the middle Lena-Aldan-Vilyui region by the Sakha-Yakut, who consequently expelled the Tungus toward peripheral regions in the mountains and beyond the Arctic Circle [25–28]. The Yukaghir culture soon faded away, and their language was superseded by Russian or by one of the Neo-Siberian languages.

We compared present-day mtDNA diversity in these groups with complete mitochondrial genomes from ancient samples from the region and placed the samples into combined genealogical trees. We used these updated genealogies to trace ancestral relationships between populations sharing sub-haplogroup-specific mutations. We used the results to reconstruct expansions (preponderantly from south to north) as well as contractions of populations, thus shedding new light on northeastern Eurasia’s past.

### Populations and samples

#### Ket

Formerly called Yenisei Ostyak (the census of 1897 recorded 988 persons), the Ket people are thought to be descendants of some of the earliest inhabitants of Central Siberia, while all of their present-day neighbors seem to be relative newcomers. In the 18^th^ century, some of the Ket were forcibly moved to the lands between the Ob and Yenisei Rivers, where the Selkup, speaking a language belonging to the Samoyed group of the Uralic language family, lived [2, 5, 19]. Until recently, there were no more than 500 Ket living in a few riverside villages in the middle reaches of the Yenisei; as in the past, many survive as seasonal hunters, trappers, and fishers. The remnants of the Southern (Upper) Ket, whose ancestors are believed to have originated from the Podkamennaya Tunguska region, have been almost completely integrated into the expanding Evenki [20].

We integrated our newly obtained mtDNA sequences from published mtDNA data [29] and collected new DNA samples from individuals residing in the villages of Turukhansk and Kellog (Turukhansk District, Krasnoyarsk Region, Russian Federation) in August 2014. The total number of Ket subjected to complete mtDNA sequencing was 34. Based on self-reported ancestry, verified by senior members of related families, we determined that the overwhelming majority of the Ket have been extensively admixed with Russians. A substantial proportion of the Ket sampled were not completely certain of their maternal ancestry in terms of Selkup or Evenki origin.

#### Tofalar

The Tofalar comprise of the remnants of several hunting tribes, gradually assimilated into a broader group. There is anthropological and linguistic supporting the classification of the Tofalar as Old Siberians [5]. Fewer than 400 Tofalar, who are reindeer breeders and hunters, inhabit the northern slopes of the eastern Sayan Mountains, along the Uda, Gutara, and Nerkha rivers. They originally spoke a Samoyed language but later changed to a language of the Turkic family. In the 17^th^ century, the predecessors of the Tofalar entered five administrative settlements of “Udinsk Land” of the Krasnoyarsk Region [2]. When the Tofalar transitioned to a settled lifestyle in 1930, they were concentrated in three newly established villages: Upper Gutara, Nerkha, and Alygdzher (Nizhneudinsk District, Irkutsk Region). In the present study, newly obtained data sampled in Alygdzher through fieldwork in 2015 were revised and integrated with those published previously [12]. In sum, the core set of Tofalar samples subjected to complete mtDNA sequencing constituted 31 individuals; the family history of 11 Udinsk Buryat suggested Tofalar maternal ancestry [12, 14].

#### Todzhi

This is a small subgroup living in Todzhinsky District in the northeastern part of the Tuva Republic encompassing the intermountain Todzhinsky Depression between the Western and Eastern Sayan. Although modern Todzhi people speak a dialect of the Tuvan language, anthropologically, they are distinct from southern and western Tuvans, being similar to the neighboring Tofalar people [21]. Census records from 2002 noted approximately 3000 Todzhi, and their traditional economy is generally considered to have had a hunting-gathering emphasis supplemented by reindeer herding and fishing. The Todzhi blood samples (n = 53) were chosen from a much larger number of samples collected through fieldwork in February 2017 in three main settlements of the Todzhinsky District (Toor-Khem, Adyr-Kezhig, and Iiy). Specifically, we selected unrelated samples from the elders born in 1962 and/or earlier for full mtDNA sequencing.

#### Yukaghir

In this revision of the maternal DNA history of the Yukaghir/Chuvantsi genetic profile, 46 samples were chosen from previously collected samples [8, 12–15], 13 of which not yet examined at the entire mtDNA genome level, and were completely sequenced (Table 1). Approximately half of the 46 complete mitochondrial genomes evidently come from individuals who were born in Markovo, Magadan Region, whereas the remaining come from the villages of Nelemnoye (Upper Kolyma district), Kolymskoe and Cherskiy (Lower Kolyma district). A portion of samples come from the villages of Chokurdakh located on the left bank of Indigirka River in its lower course and Andrushkino on the Alaseya River (Allaikh district, Sakha-Yakut Republic). Notably, a core of the Alaseya Yukaghir is made up of the Tungus people who came from the lower reaches of the Lena River and married into Yukaghir [30].

**Table 1:** List of mtDNA (sub)haplogroups found in the dataset and their frequencies in four populations.

	Ket (34)	Todzhi (53)	Tofalar/Ud. Buryat (42)	Yukaghir/Chuvantsi (46)
A8a2	2		1	
B4a1c2		1	1	
B4d			1	
B4j		1		
C4a1a-195!			3	
C4a1a3		4	6	2
C4a2a1	4	1	7	1
C4b		8	2	10
C4b1		9	2	1
C4b3a		2	1	3
C4b7				6
C5a1		6		
C5a2b				3
C5b1a		3	4	
C5d1		1		4
D4b1c/D3				4
D4b1a2		2		2
D4e4				1
D4i2				1
D4j	1		2	2
D4l2a				2
D4m2				1
D4p		2		
D5a2a		1		
F1b1b	1	8	1	
F1b1e			1	
H101*	1			
H1ae3*	1			
H1b2	1			
J1c2m	1			
G1a1		1		
G2b2		1		
M8a3			7	
N2a	2			
U3b		2		
U4a1	8			
U4d2	3			
U5a1d2	3			
U5b2b			1	
Y1a1	4			
Z1			2	
Z1a	2			3

## Results

Hereafter, we describe the genetic diversity of complete mtDNA sequences from the Ket, Tofalar, Todzhi, and Yukaghir and compare them with previously reported data. We also combine these data with new mtDNA from the Mansi, Tubalar, Nganasan, Evenki, Even, Chukchi and Koryak, many of whom were sequenced at the full mtDNA genome level, building on smaller amounts of data initially reported from these samples [12–15, the study herein]. Table 1 presents the distribution of well-defined lineages within and between 4 groups. The approximate location of 218 mtDNA complete sequences, along with accession numbers in GenBank, are given in S1 Table in S1 File.

Note that these are haplogroup frequencies, and individuals belonging to the same subhaplogroup might have distinct haplotypes. The summed number of individuals in the column is presented in parentheses.

### Major mtDNA lineages in their Eurasian context

#### Haplogroup N2a

**Fig 2:**
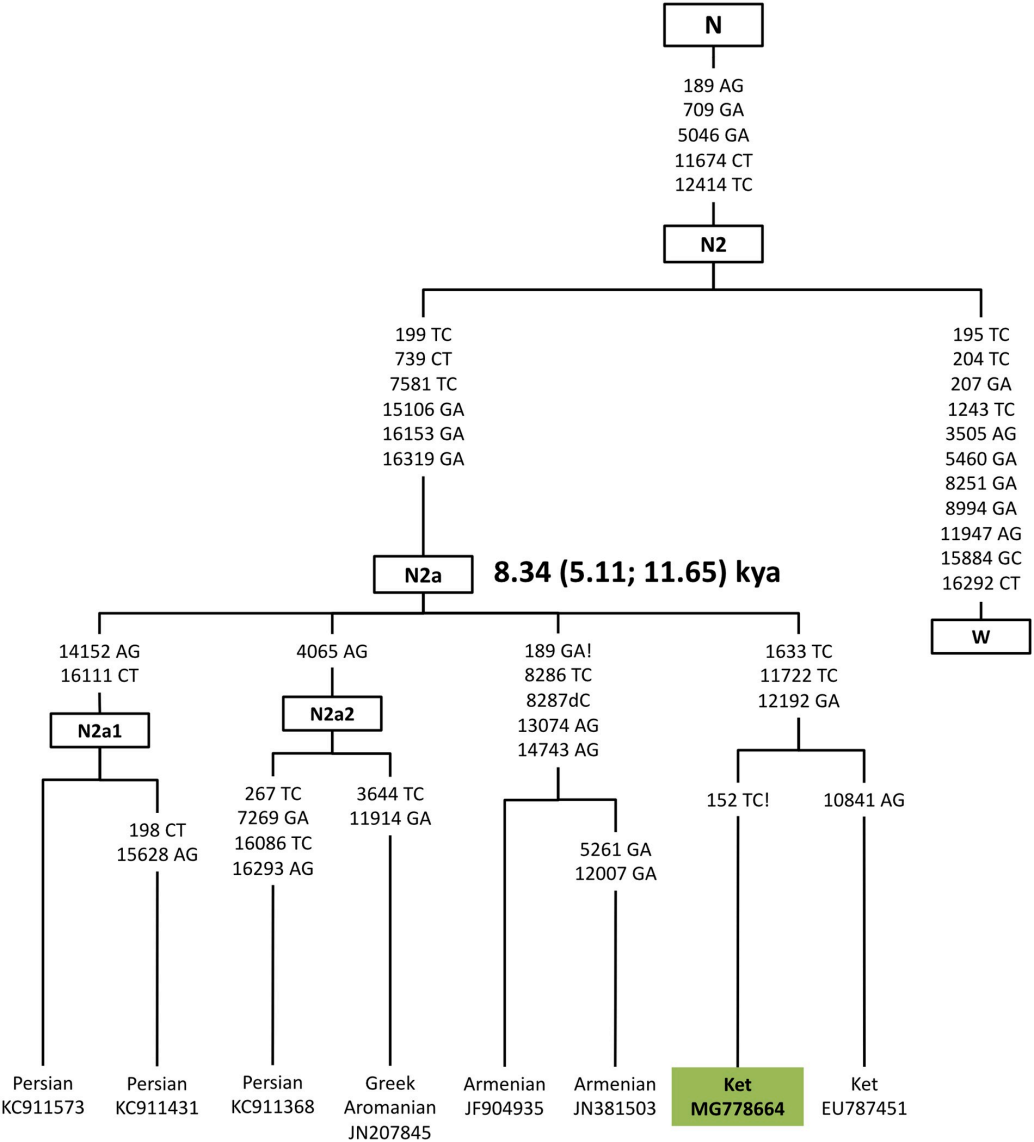
A phylogeny of complete haplogroup N2a sequences. General legend for phylogeny figures: Bold and green text indicates new sequences generated in this study. When two or more identical samples belong to the same group, their numbers are given in brackets. Bold and yellow text indicates ancient sequences newly generated in this study, and gray indicates ancient sequences obtained from published sources (see S2 Table in S1 File). Dashed lines for ancient samples indicate that the sequence is not complete and contains gaps due to DNA damage or contamination. Haplogroups’ age estimates in thousands of years are presented in bold.

The Yenisei region was located outside the main routes of Eurasian agricultural exchange up to the time of the Late Bronze Age Karasuk Culture [31–33]. One of the remarkable features of the Ket mtDNA pool is a lineage of haplogroup N2a distinguished by a set of mutations that we newly document here (m.1633T>C, m.11722C>T, and m.12192G>A) (Fig 2). Whereas the previously published N2a sample uniquely marked by m.10841A>G (EU787451) is from the Upper Ket in the village of Sulamai on the Stony Tunguska River [29], our newly identified N2a mitogenome lacks this mutation and comes instead from the village of Kellog in the Lower Ket. Apart from the Ket individuals, none of the extant or ancient Siberian populations sampled to date are known to carry N2a. This haplogroup is found in just a few contemporary individuals from Europe, Iran, Arabia, and Ethiopia [34, 35]. It is likely that this lineage came to the mid-Yenisei from the Caucasus, presumably the major source of N2a, which contributed to the central Siberian maternal lineages.

#### Haplogroup U4a/U4a1

**Fig 3:**
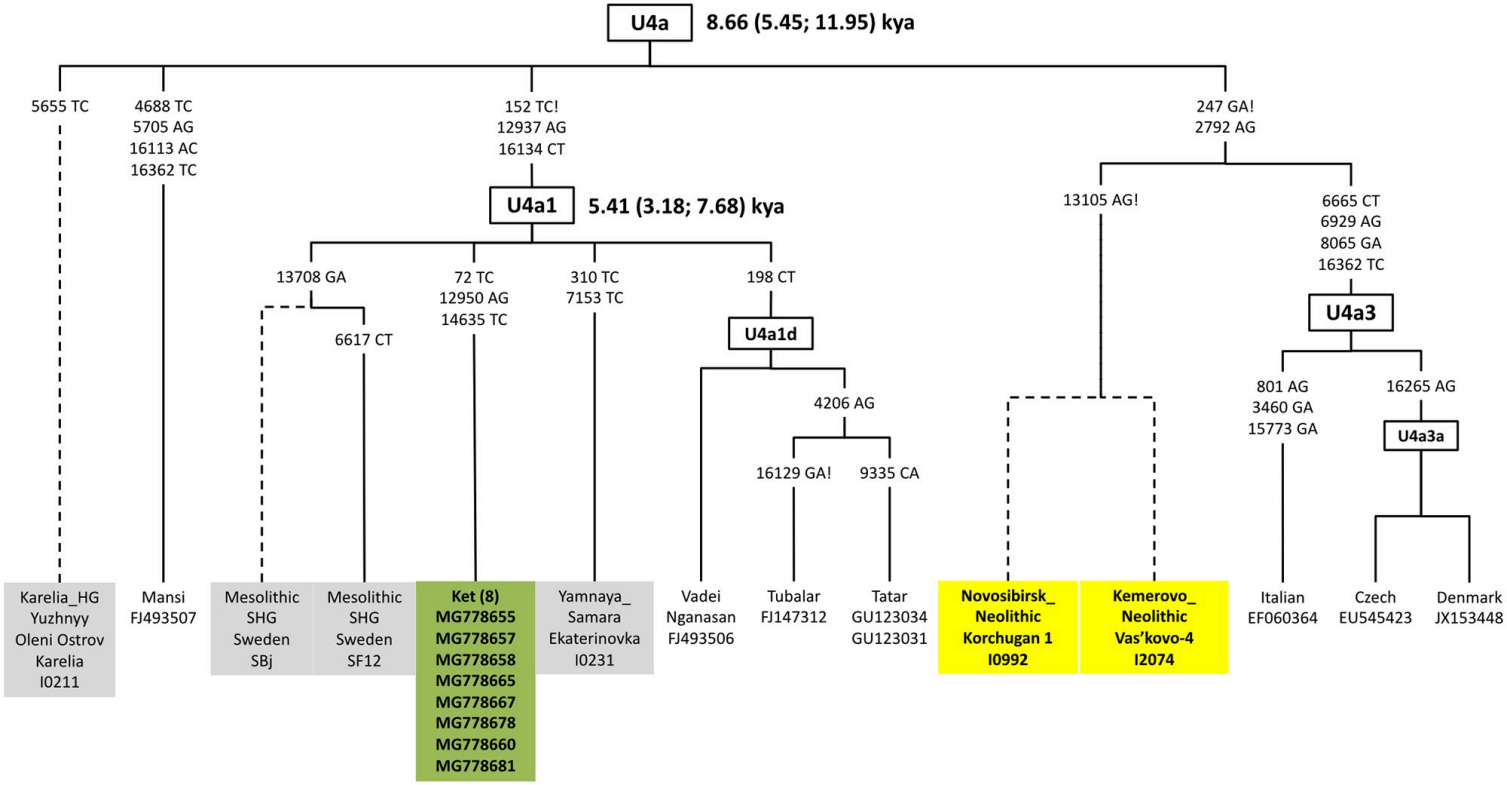
A phylogeny of distinct U4a branches and subbranches.

Haplogroup U mtDNA, confined to subhaplogroups U2e, U2e1, U3, U4a, U4b, U4c, U4d, and U5a1, is well represented in Old Siberian groups in the northern Altai-lower Ob-Yensei triangle [13, 14, 23, 29, 33, 36] including in our newly reported data. We built a phylogenetic tree of the U4a/U4a1 subhaplogroups, including ancient mtDNA sequences and their Ket counterparts, or those from the regions close to the Ket traditional territory (Fig 3). The entire tree, combining the Ket, Tubalar, Mansi, and Vadei Nganasan sequences with ancient mitochondrial genomes, a newly reported genome from Novosibirsk (I0992: 5002–4730 cal BCE), and a newly reported genome from the neighboring Kemerovo region (I2074: 5602–5376 cal BCE), suggests that the U4a1 lineage was present by Neolithic times in Altai-Sayan, where it could have diversified in situ, with U4a1 and U4a3 dispersing into the heartland of Europe. The complete U4a1 mtDNA sequence in the Yamnaya (I0231) [37] culture (the Beaker people of western Europe in the early Bronze Age represented the far western extent of Yamnaya ancestry) spread from Samara region and Mesolithic genomes from the Sweden is consistent with the association of the U4a lineage in West Siberia with Mesolithic ancestry.

#### Haplogroup U4d

**Fig 4:**
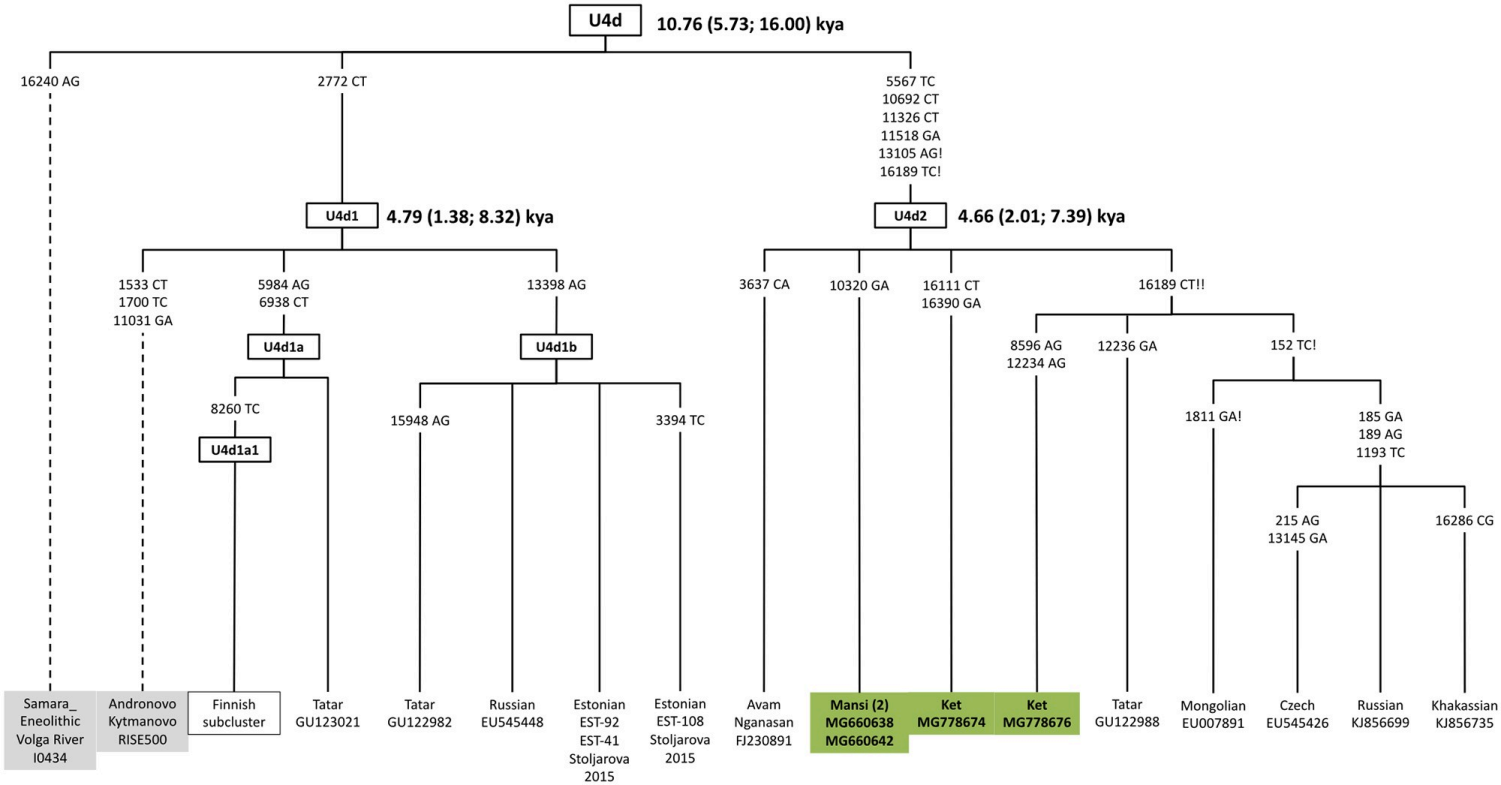
A phylogeny of complete U4d sequences compared with a couple of ancient (incomplete) sequences.

The updated phylogeny of U4d includes both its main subhaplogroups U4d1 and U4d2 and three previously unreported sublineages documented in two Ket and two Mansi mitochondrial genomes (Fig 4). Whereas the U4d1 sequences are prevalent in the Baltic Sea region, the U4d2 haplogroup is associated with various tribes in areas where eastern Uralic speakers [13] expanded. Specifically, the observation of the U4d1 sequence in a Bronze Age individual (RISE500) from Kytmanovo (along the Chumysh River in the northwestern Altai) supports the hypothesis of an east-to-west pattern of divergence of Uralic languages [22, 38]. The coalescence time of the U4d1 and U4d2 subclusters (~4.7 kya) is consistent with the hypothesis of continuous gene flow between the Yenisei River valley and the eastern Baltic region during and following the Bronze Age [14]. This conclusion is in agreement with ancient genome-wide data from Finland and the Russian Kola Peninsula [39], revealing that the specific genetic makeup of northern Europeans reflects important ancestry from Siberian migration that began at least 3,500 years ago [24].

#### Haplogroup U5a

**Fig 5:**
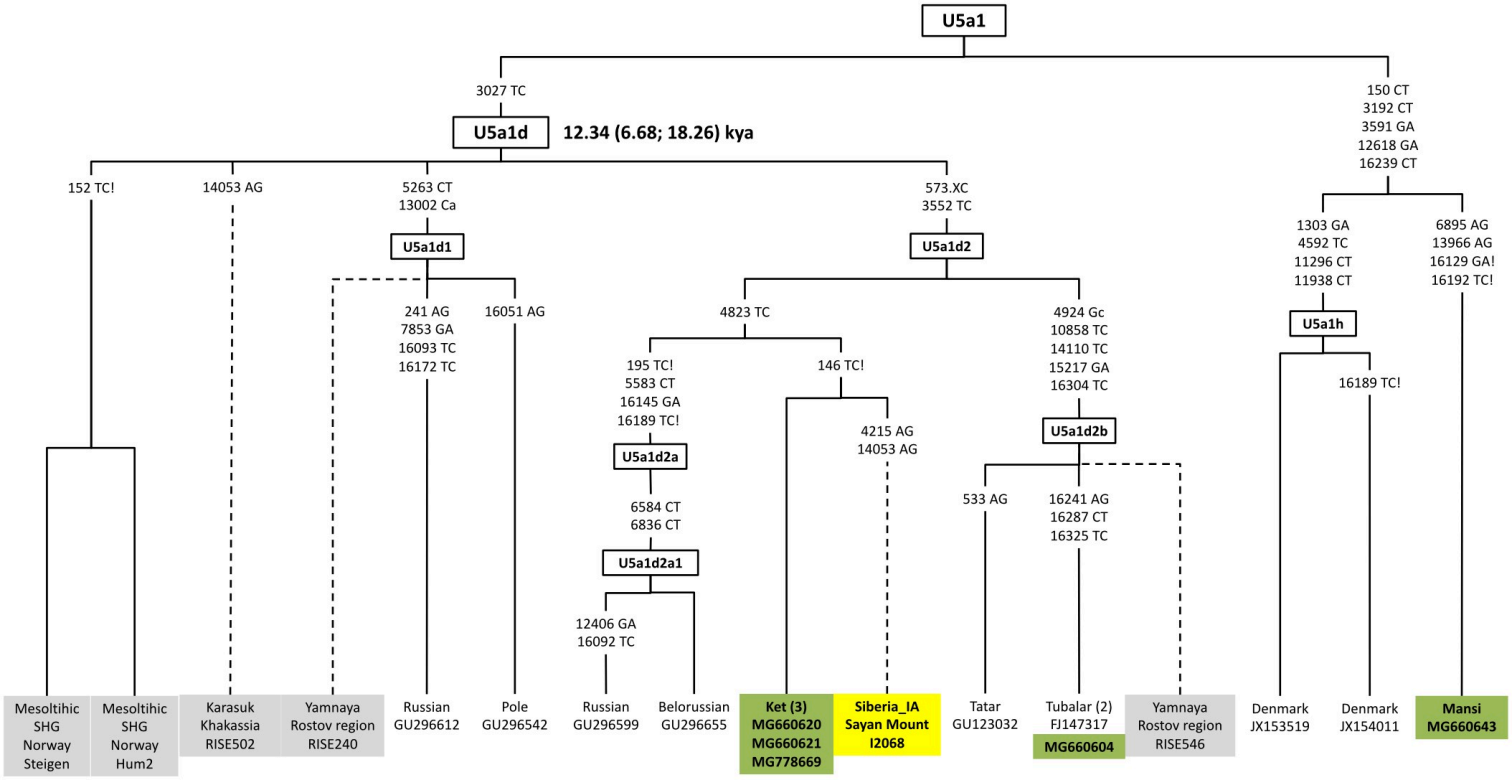
A phylogeny of U5a1 revealing distinct branches or subbranches in the Ket, Tubalar, and Mansi mitochondrial genomes compared with ancient sequences.

Recent studies utilizing the genome-wide approach suggest that U5a lineages already existed in Mesolithic Fennoscandia (U5a1 and U5a2) and may imply an eastern origin of these sublineages in Europe [40, 41]. Three of our new Ket sequences clustered into U5a1d2 with a newly reported ancient mitochondrial genome (I2068: 420–565 cal BCE) from the Yenisei-Sayan area, whereas two of the Tubalar sequences fell into U5a1d2b. Previously unrecognized haplogroup U5a2 haplotypes (U5a2b and U5a2a1b) were identified in a few Mansi mtDNA samples chosen from a pool of previously collected samples [42]. With the novel U5a1h haplotype harbored by a Mansi individual, these findings revise our understanding of U5a1 haplogroup relationships (S1 Table in S1 File, Fig 5).

#### Haplogroup A8

**Fig 6:**
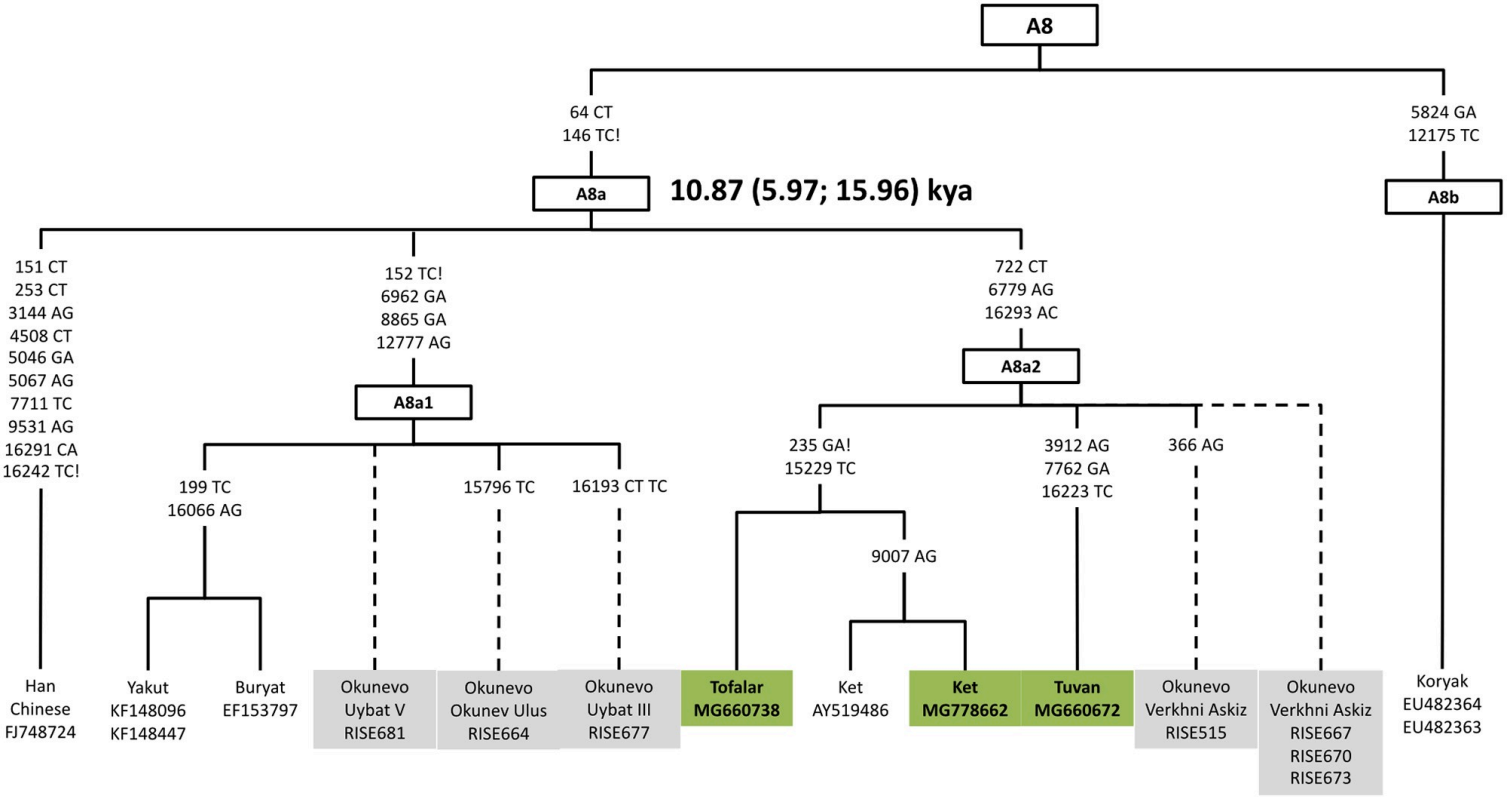
A phylogeny of A8 revealing distinct lineages or sublineages.

In this study, previously published and newly obtained A8a2 samples were explored to redefine the structure of the entire A8 tree (S1 Table in S1 File, Fig 6). As a result, we were able to delineate a previously uncategorized A8b lineage [43]. The immediate split of haplogroup A8 created a distinctive A8b lineage evident in two Koryak mitochondrial genomes. The entire tree expands our understanding of haplogroup A8 by encompassing ancient mitochondrial genomes, recently attributed to the Bronze Age Okunevo culture by Pilipenko et al. 2018 [44].

#### Haplogroup M8a3

Seven of the Tofalar mtDNA samples, initially termed haplogroup S [12], fell into the M8a3 haploroup (Table 1, S1 Table, S1 Fig in S1 File). The origin of the M8 root appears somewhere within Southeast Asia, subsequently splitting into the M8a and CZ clusters in the area currently inhabited by modern East Asians [12, 45, 46]

#### Haplogroups C4a1 and C4a2

When assembled into one phylogenetic tree with their ancient counterparts, haplogroup C4a1 haplotypes, common in the Tofalar, Todzhi, Evenki, Even, and northern Altaian (Kumandin, Chelkan, and Teleut), redefine the ancestral C4a1 types. At their widest extent, the carriers of C4a1 lineages and sublineages spread from the Himalayas, Mongolia, and Iran across the south-central Siberia to Chukotka (S3 Fig in S1 File), contrasting with the pattern observed for C4a2 diversity, which is basically restricted to C4a2a (S4 Fig in S1 File).

#### Haplogroups C4d and C4e

The age estimates of the newly described sublineages hint at the origin and diversification of the C4 haplogroup in Siberia/Asia (S2 Fig in S1 File). Thus, modern samples (C4d and C4e) nested in the C4-m.152T>C node together with the newly reported ancient individual I2072 (Solontsy-5 from Altai, dating to 3959–3715 cal BCE) and RISE602 (Sary-Bel, 900 BC– 1000 AD) samples. Likewise, the clustering of I1000 from Obkhoy, from the Glazkovskaya culture and dated to 2871–2497 cal BCE, in C4 supports ancient gene flow within the southern extent of the Central-Eastern Siberia. The coalescence time estimate of C4-m.152T>C-m.16093T>C was approximately 20 kya. Unlike the C4e subhaplogroup revealed in northwestern Altai, the C4d lineage found outside Siberia shared common ancestry with the Tibetans or populations currently living with Tibetans [47, 48]. On the other hand, C4c, a sister group of C4a and C4b [13] is found solely in the Western Hemisphere. The present-day C4c distribution in North America is centered on the southern end of the former glacial ice-free corridor. If this is a significant signature of its early dispersal history, the haplogroup could have been carried by the earliest peoples of central North America, who are argued on genetic [49] as well as archeological and paleoecological grounds to have expanded southward from Beringia/Siberia into America sometime before 13.5 kya [50, 51].

#### Haplogroup C5

The phylogeny of C5 is structured into four major branches, assigned as C5a, C5b, C5c, and C5d, in accordance with the latest release of PhyloTree (S5-S7 Figs in S1 File). While the C5a1 and C5a2a samples are from the Altaic-speaking populations scattered across the southern extent of Eastern Siberia, the C5a2b is likely to reflect different migrations of Reindeer Koryak, Chukchi, Yukaghir, and Kamchatkan Even. An updated C5-m.16093T>C network, including 29 entire sequences, of which 15 are new, is given in S5 Fig in S1 File. Accordingly, the entire tree splits into two main lineages: C5c and C5d. While the C5c-m.16291C>T lineage is confined largely to the Teleut and Kumandin from southwestern Siberia, C5d1 is associated with the groups spread in the area where the Tungus-speaking populations expanded. Interestingly, C5d, which is encountered in low frequency in the Tuvan/Todzhi, Evenki, Even, and Yukaghir, was recently retrieved from 8,400-year-old sample in Inner Mongolia by Wu et al. 2019 [52], implying imperceptible genetic admixture and indicating continuity between ancestral populations of East Asia and northeastern Siberia (S1 and S2 Tables in S1 File, Fig 4). In addition to Okunevo samples previously reported from West Siberia [53], ancient samples of this type were collected in the Transbaikal; one is Mesolithic (irk00x), and the other is pre-Bronze Age (irk078) [54].

The tree of haplogroup C5b (S7 Fig in S1 File) is conspicuous because of the C5b1a1 sublineage harbored by 4 of 39 (10.3%) Nganasan, 4 of 40 (10.0%) Tofalar, and 3 of 51 (5.9%) Todzhi individuals. The entire picture indicates a relatively recent affinity of the Nganasan from the Taimyr Peninsula to the Todzhi and Tofalar peoples. This finding is congruent with phylogenetic analysis of autosomal HLA class II genes in the respective populations. In all the neighbor-joining trees based on gene frequencies at HLA class II loci, the Nganasan cluster together with the Tofalar and Todzhi populations [55, 56], thus corroborating a close genetic link between reindeer hunters of the Taimyr Peninsula and reindeer breeders of the eastern Sayan Mountains.

#### Haplogroup Z1

A novel Z1 sublineage, designated here as Z1b and formed by two Tofalar samples, with one haplotype from western Sayan [12] and another from eastern Sayan (this study), is noteworthy (S8 Fig in S1 File). An ancient sequence falling within the same sublineage of Z1, harboring new m.9804G>A, m.10295A>G, and m.15172G>A, was identified at the Korchugan site from the Novosibirk region. This sequence, which was recovered from an individual dated to 5206–4805 cal BCE (I0991), must have arrived in Central Siberia. We also document an instance of Z1a1 in the nearly reported ancient sample I0998 (Khuzhir site from Olkhon Island in Lake Baikal, 2835–2472 cal BCE). An entire tree features Z1 coalescing at ~11.8 kya, whereas the Z1a node is much younger, dated to ~4.9 kya. Distinct sublineages of the Z1a haplogroup and their derivative haplotypes harbored by the Tubalar, Tofalar, Nganasan, Yukaghir, Koryak and Tungusic-speaking Evenki, Even, and Ulchi could represent a major part of Neolithic dispersals from southeastern Siberia, with Z1a1a emerging in the present-day Ket, Volga-Urals, Finns and Saami much more recently. The Z1a1b haplotypes detected in single carriers, referred to previously as Nganasan or Yukaghir [11, 12], in fact may be of Tungusic origin due to relatively recent gene flow from nearby Evenki or Even groups that have patchily inhabited Arctic Siberia since early the period of Russian expansion.

#### Haplogroup F1b1

Of the 53 mitochndrial genomes sampled from Todzhi, 8 (15.1%) fell into two different sublineages of the F1b1b haplogroup (S1 Table, S9 Fig in S1 File). Accordingly, the phylogeny of F1b1b is structured into three major subbranches, assigned as F1b1b1, F1b1b2, and F1b1b3. It is not surprising to observe the Ket identity of F1b1b2, confirmed by sharing of the m.10227T>C and m.16179C>T variants, which was revealed earlier in 23.7% of Ket restricted to Podkamennaya Tunguska [29]. Remarkably, F1b1b2 was identified in a Late Bronze Age individual from Afontova Gora on the left bank of the Yenisei River (RISE553), and F1b1b3 was identified in another, roughly contemporaneous sample from the same site and archeological culture (RISE554). Recently, older samples (F1b1b) were described with deeper, pre-Bronze Age time depths in the region west and north of Baikal Lake (irk036 and irk068) [54].

## Discussion

### Postglacial recolonization of northeastern Siberia

An unresolved issue in understanding the postglacial resettlement of the Siberian Arctic is population substructure before the era of pastoralism, which emerged in the form of reindeer herding after ~1200 CE [51] and could have had significant impacts on the population-genetic landscape of this vast region. The autochthonous Paleoasiatic hunters, before the disruption caused by reindeer breeders’ massive colonizations, may be seen as a link in the gradual cultural (and in some cases linguistic) continuum connecting the Uralic-, Yeniseian-, Yukaghir-, Amur-, and Eskaleut-speaking groups [57–59]. Interestingly, the Entsi/Nganasan people retained a lifestyle comparable to and arguably continuous from that of prepastoral reindeer hunters making the Taimyr Peninsula the last refuge for a palimpsest of reindeer hunters, the least touched genetically by modern herding groups as recently as the 1950s [2, 13, 60–62]. A major complication in studying the genetic prehistory of Yukaghir tribes is profound admixture with Neo-Siberians, Tungus-speaking populations and (to lesser extent) the Yakut [13, 45, 63]. This admixture is a challenge for learning about the historical relationships among the Yukaghir and/or proto-Yukaghir populations. To overcome this complication, we focused on the analysos to mtDNA attributed to Yukaghir-specific haplogroups, mainly C4b and D4b1c/D3. The pattern of the basal branches of C4b detected among the modern and ancient inhabitants sampled from the Yana-Indigirka-Kolyma-Anadyr interfluvial (S10 Fig in S1 File) suggests that the initial episodes of C4b and D4b1c/D3 diversification occurred in the Lena/Amga/Aldan area (south-central Yakutia) in the Neolithic, after which the groups dispersed via geographic expansion during and after the Bronze Age. This conjecture is supported by an exceptionally large variety of C4b sublineages sharing C4b roots (m.3816A>G) in populations with different linguistic affiliations [10, 12–15, 45, 64]. The interaction between reindeer Koryak, Chuvan, Khodyn and Anaul (the latter two tiny tribes dissolved among the Chuvan (Chuvantsi) shortly after the first Russians appeared in Chukotka in the mid-17^th^ century) are consistent with historical records indicating that quite a few Yukaghir/Chuvantsi women were among the Koryak and the Chukchi by the end of the 19^th^ century [6, 45]. We also highlight the evolutionary history of D4b1c/D3, whose intrinsic diversity has not yet been well resolved [13]. Following increased sampling, the haplogroup D4b1c/D3 phylogeny encompasses seven Nganasan and four Yukaghir, which together account for 47.8% of the entire D3 sample tested at the complete mitochondrial genome level (S1 Table, S11 Fig in S1 File). Furthermore, the detection of D4b1c/D3 in samples irk022 (2455–2200 cal BCE) of the Bronze Age from Cis-Baikal and N5a (4340–4235 cal BCE) of the Middle Neolithic from Yakutia, recently determined by Kılınç et al. 2018 [54], provides additional support for the Nganasan/Yukaghir/Chuvan association. The co-represention of C4b and D3 in the Neolithic mtDNA genomes sampled from Yakutia (with high confidence) [50], suggests that the ancestry of the Yukaghir is multipartite and derived from the area that encompassed the East Sayan, Lena/Aldan valleys, and the northwestern vicinity of Lake Baikal. The Yukaghir ancestors were apparently interrupted by the expansion of the Tungus-speaking people who arrived from the Siberian taiga but whose original homeland was the area around Lake Khanka in the southern part of Primorye [65].

## Conclusions

Here, we have assembled novel data on unique native Siberian populations, with a focus on the Ket, Tofalar, Todzhi, and Yukaghir. The whereabouts of the Ket and Yukaghir population histories are long-standing issues. The new mtDNA data have been critically reviewed to provide a clearer picture of the number and timing of founding lineages. Our findings reveal the origins and expansion histories of mtDNA lineages that evolved in south-central Siberia bordering Central-Inner Asia, as well as multiple phases of connections between this region and distant parts of Eurasia. Our results illustrate the advantage of jointly sampling complete modern and prehistoric mitogenomes to fully measure past genetic diversity and to reconstruct the process of peopling at high latitudes on the Siberian subcontinent.

## Methods

### Ethics statement

Blood samples were collected from aboriginal Siberians with appropriate informed consent (written) during multiple expeditions conducted, mainly, by Rem Sukernik and Elena Starikovskaya in 1993–2017. The protocol and the collection of samples was approved by the Ethics Committee of the Animal and Human Research Section, Institute of Molecular and Cellular Biology, Siberian Branch, Russian Academy of Sciences (protocol №01/19, 21.01.2019). Written consent to use their samples was obtained from all donors after explanation of the aims of the study. The research has been performed in accordance with the WMA Declaration of Helsinki (59th WMA General Assembly, Seoul October 2008). All methods were carried out in accordance with the approved guidelines and regulations.

### Mitochondrial genome sequencing of modern samples

Genomic DNA was extracted from blood buffy coats using standard procedures. The complete sequencing procedure of modern samples entailed PCR amplification of 2 overlapping mtDNA templates, which were sequenced with an Illumina HiSeq 2000 instrument [Illumina Human mtDNA Genome Guide 15037958B]. Short reads were analyzed with the BWA-backtrack tool [66] and Unipro UGENE version 1.21 software [67].

#### Archelogical context for newly reported ancient samples

In a dedicated clean room at Harvard Medical School, we prepared powder from the teeth of 7 individuals, all of whom we directly radiocarbon dated using accelerator mass spectrometry. These individuals consisted of the following:

- I0991 (Korchugan1-3, Novosibirsk, Korchugan 1; 5206–4805 cal BCE (6060±50 BP, Poz-83427)),
- I0992 (Korchugan1-7, Novosibirsk, Korchugan 1; 5002–4730 cal BCE (5990±50 BP, Poz-83428)),
- I0998 (Khuzhir-2, Irkutsk, Olkhon Island, Lake Baikal, Khuzhir; 2835–2472 cal BCE (4040±35 BP, Poz-83426)),
- I1000 (Obkhoy-7, Irkutsk, Kachugskiy, Obkhoy; 2871–2497 cal BCE (4100±40 BP, Poz-83436)),
- I2068 (230/3, Kurgan 2, Sayan Mountain, Minusinskaya Intermountain Basin, Tepsei III; 420–565 cal CE (1560±30 BP, Poz-83507)),
- I2072 (230/13, Altai foothills, Solontcy-5; 3959–3715 cal BCE (5050±40 BP, Poz-83497)), and
- I2074 (230/18, Burial 1, Vas’kovo 4, Intermountain basin between spurs of Altai and Sayan Mountains; 5602–5376 cal BCE (6520±40 BP, Poz-83514)).

Ancient DNA was generated according to previously established methodology, so we do not discuss the procedure in detail here [68]. Briefly, we extracted DNA [69] and converted it into individual barcoded double-stranded libraries in the presence of uracil-DNA glycosylase (UDG) and Endo VIII (USER, New England Biolabs) to characteristic errors at all but the final nucleotide [70]. We enriched the libraries for sequences overlapping the mitochondrial genome [71] and then sequenced them on an Illumina NextSeq 500 instrument using v.2 150 cycle kits for 2×76 cycles and 2×7 cycles. We computationally removed the barcode and adapter sequences and merged pairs of reads, requiring a 15 base pair overlap (allowing up to one mismatch). We mapped the merged sequences to the reconstructed human mtDNA consensus sequence [72] using bwa (v.0.6.1) [66]; removed sequences with the same strand orientation and start and stop positions; and built a consensus mitochondrial genome sequence for each sample (average coverages were 25-1550-fold). We used contamMix-1.0.9 to estimate 95% confidence intervals for the rates of mismatch to the mitochondrial DNA consensus sequence, all of which were fully contained in the range 0.99–1 [73]. All samples had a rate of damage at the final nucleotide in the range of 0.028–0.127.

All specimens analyzed in this study were obtained from Repository of paleoanthropological collections of Institute of Archaeology and Ethnography SBRAS (Novosibirsk, Russia) and provided by Anatoly P. Derevianko, one of the co-authors of this manuscript. No additional permits were required for the described study.

#### Mitochondrial data analysis

All mtDNA genome consensus sequences were called using SAMTOOLS mpileup [74]. The resulting consensus sequences were then inspected by eye, with particular attention being paid to the hypervariable regions and nucleotide positions previously identified as being problematic [43]. All ambiguous sites were called as ’N’. Mitochondrial haplogroups were assigned with mitohg v.0.2.8 software [75].

Entire mtDNA sequences were assembled into phylogenetic trees by using the median-joining algorithm of Network 4.5.1.024 [76] and mtPhyl v5.003 software (http://eltsov.org). Coalescence dates were estimated with the ρ statistic [77]. Standard errors (σ) were calculated according to Saillard et al. 2000 [78]. Mutational distances were converted into years using the substitution rate for the entire molecule, 2.67×10^−8^ substitutions per site per year [73]. The haplogroup affiliations reported in this analysis correspond to the current nomenclature of mtDNA in agreement with the latest release (February 2016) of PhyloTree Build 17 [43].

Overall, 218 newly reported mitochondrial genomes are listed in S1 Table in S1 File along with corresponding ethnicities, sample locations, and GenBank accession codes. In this study, 70 previously published ancient mtDNA sequences were used to provide powerful new information about subhaplogroup affiliation (S2 Table in S1 File) [32, 33, 37, 41, 53, 54]. To uncover ancient mtDNA lineages, especially those from the Altai-Sayan area, we compared modern mitogenomic data with their ancient counterparts sampled from skeletons recovered from the Euro-Siberian region that extends from Central Europe and Scandinavia to Lake Baikal.

## Supporting information

S1 File (ZIP)
