# Supplementary material for: Mitochondrial genome diversity on the Central Siberian Plateau with particular reference to the prehistory of northernmost Eurasia
Source: PLoS One. 2021 Jan 28;16(1):e0244228. doi: 10.1371/journal.pone.0244228 (PMC7842996; doi:10.1371/journal.pone.0244228)
Supplement: S1 File — (ZIP) [file pone.0244228.s001.zip › S1_File.pdf]

Supplementary Information

Mitochondrial Genome Diversity in the Central Siberian Plateau with Particular Reference to Prehistory of Northernmost Eurasia

S. V. Dryomov, A. M. Nazhmidenova, E. B. Starikovskaya, S. A. Shalaurova, N. Rohland, S. Mallick, R. Bernardos, A. P. Derevianko, D. Reich, R. I. Sukernik.

Samples from South Siberian archaeological sites

Korchugan-1

Human fossils from the Neolithic cemetery located in the pre-taiga zone of the West Siberian Plain (Kyshtovka Region of Novosibirsk Province; N 56°28′; E 76°18′). Koshkurgan-1, a burial yard with earthen mounds, were excavated by Academician V.I. Molodin in 1996. Available calibrated radiocarbon dates indicate an age of 6th – 5th centuries BCE. Three tombs were attributed to the Neolithic period; human fossils from burials 3 and 7 are the focus of the work here. The three Neolithic tombs form a row oriented along the NW-SE line. The complete information is provided in the paper "The Neolithic burial ground of Korchugan in the Middle Tara" by V.I. Molodin, A.V. Novikov, T.A. Chikisheva in the book "Neolithic-Charcolithic Period in southern West Siberia. Kemerovo: Izdat. Kuzbassvuzizdat, 1999, pp. 66 – 98."

I0991 (Korchugan1-3)

Burial 3 was established in an ovoid grave, 2.2 x 0.75 x 0.25-0.17 m in size; the grave floor was uneven with one of the short sides higher than the opposite one. The occupant is an adult male of 45 – 50 years of age, large in stature, and the features of the bones testify to well-developed muscles. The body was placed in the tomb in the stretched supine position with the head towards N-NE.

I0992 (Korchugan1-7)

Burial 7 was established in the grave, 2.23 x 1.05 x 0.5-0.64 m in size. The occupant was estimated as a female 17-23 years of age. The body was placed in the tomb in the stretched supine position with the head toward the N. The hands were placed over the hip bones. Pendants of squirrel (?) canine teeth were found close to the right knee. A necklace of nine miniature flat bird figurines made of bone with hanging openings was found over the chest. The tenth element in the necklace was a tear-shaped bone pendant.

Craniometric and the long bone measurement data from the Neolithic burials at Korchugan-1 are provided in the abovementioned paper.

The features of Korchugan-1 burial rite are similar to those noted at the Neolithic sites in the Ob basin and likely represent a single historical-cultural unity. However, the area was populated by anthropologically diverse populations. The Korchugan-1 materials represent an anthropologically distinct group related to the population of the Altai Plain and Kuznetsk Hollow.

I2072 (230/13 – Solontsy-5, tomb1)

Tomb 1 was found in the earthen mound yard of Solontsy-5 on the terrace remnant on left side of the Biya at the confluence with the Chepshushka: N 52°29'; E 86°13'.

The site is situated in the forest-steppe zone of the Altai-Salair piedmonts. Excavations were carried out in 2000 – 2001 by Natalia Y. Kungurova. The age of the site was estimated as

4850–4502 BCE, and the burial features attest the Kuznetsk-Altai archaeological culture. All the nine tombs of the site were oriented along the NW – SE line. The dead were placed in the tombs in the stretched supine position, the arms were stretched along the body, the heads were oriented towards the NE-E.

The sample represents the upper medial canine tooth of an adult individual – male (?) of an estimated age of 35 – 40 years. Remains of an infant, which age was estimated to approximately 18 months basing on the dental system features and the size of the femurs was found in the same tomb. The skull is poorly preserved due to the post-mortem deformation. The skull is moderately developed, and the mandible is relatively gracile. The estimated stature varies from 161.7 cm to 166.5 cm depending on the technique.

I2074 (230/18 – Vas'kovo-4, tomb 1)

The Vas'kovo-4 (N 55°03′; E 85°05′) is an earthen burial mound yard located on the high right bank of the Tykhta close to its junction with the Inia in the vicinity to the village of Vas'kovo. The cemetery was studied by Y.M. Borodkin in 1967; tomb 1 was excavated by V.V. Bobrov in 1979. The burial features attest the Kuznetsk-Altai archaeological culture. The deceased was placed in the tomb in the supine position with the head towards N – NE. The site is situated in the Kuznetsk Hollow in the forest-steppe zone of the Altai-Salair piedmonts.

Osteological analysis indicates an adult male (Maturus). The sample for genetic analysis represents the second upper premolar.

I2068 (230/3 – Tepsei-III, mound 2)

A cluster of cemeteries of various historical-cultural periods (N 53.96; E 91.56) was situated at the Tepsei Mount on the Yenisei right-side bank at the confluence with the Tuba at 25 km northwards from Minusinsk. Rescue archaeological work was carried out at the site under the project of the Sayan-Shushenski Electric Power Station construction in 1968 – 1970. The burial sites were attributed to the Afanasievo culture of the Charcolithic Period until the Yenisei Kyrgyz Culture of the early medieval period. Mound 2 was initially attributed to the Afanasievo Culture; the features of the burial rite were not published. The radiocarbon date of 420-565 calCE (1560±30 BP, Poz-83507) indicate the period of the Tashtyk Culture.

The sample for genetic analysis represents the upper molar of an adult male of 30 – 40 years old.

I0998 (Khuzhir-2, tomb 2)

The tomb was located in the northwestern part of the Olkhon Island in Lake Baikal (N 53.19; E 107.34). The site was excavated by A.P. Okladnikov in 1992. The grave (280 cm long and 65 cm wide) was covered with stone pavement, overlain by soil and turf. The tomb was damaged by looters. The bones of a male of 35 – 40 years old were scattered over the grave. The hands and feet were in situ indicating the stretched supine position of the dead. The site was attributed to the Serovo Culture. The sample for genetic analysis represents the second upper premolar.

I1000 (Obkhoy, tomb 7)

The burial yard was located close to the village of Obkhoy on the northwestern bank of Lake Baikal (N 54.02; E 105.47). Tomb 7 was excavated by A.P. Okladnikov in 1971. Human remains indicating a young male of 30 – 35 years old were found in the stretched supine position

with the head towards the NW. The burial was attributed to the Glazkovo Culture. The sample for genetic analysis represents the second upper premolar.
