# Supplementary material for: Mitochondrial genome diversity on the Central Siberian Plateau with particular reference to the prehistory of northernmost Eurasia
Source: PLoS One. 2021 Jan 28;16(1):e0244228. doi: 10.1371/journal.pone.0244228 (PMC7842996; doi:10.1371/journal.pone.0244228)
Supplement: S1 File — (ZIP) [file pone.0244228.s001.zip › S1_Table.pdf]

Supplemental Table S1. List of modern mtDNA samples generated in course of this study.

#	Haplogroup	Ethnicity	Sampling location	Genbank ID	Distinguishing / Private mutations
1	A10	Mansi	Shaim	MG660641	C64T, G2831A, A16230G!, C16256T
2	A14	Tuvan	Bai-Tal	MG660670	A4123G, A8396G, T11253C, T16086C
3	A8a2	Ket	Kellog	MG778662	G235A!, A9007G, T15229C
4	A8a2	Tuvan	Bai-Tal	MG660672	A3912G, G7762A, T16223C
5	A8a2	Tofalar	Alygdzher	MG660738	G235A!, T15229C
6	A8b	Koryak	Karaginskiy district	MK217926	-
7	B4a1c2	Todzhi	Iiy	MG660688	A16183C
8	B4j	Todzhi	Toora-Hem	MG660675	8281del10!, A15172G!, A16183C
9	C4a1a-195!	Tofalar	Alygdzher	MG660724	A14878G
10	C4a1a-195!	Tofalar	Alygdzher	MG660726	A14878G
11	C4a1a-195!	Tofalar	V. Gutara	MG660743	A14878G
12	C4a1a3	Evenki	Chumikan	MG660612	-
13	C4a1a3	Koryak	Karaginskiy district	MK217925	-
14	C4a1a3	Ud. Buryat	Kushun	MG660624	G6852A, T7954C, A13857G, (T16093C)
15	C4a1a3	Ud. Buryat	Kushun	MG660628	G6852A, T7954C, A13857G
16	C4a1a3	Ud. Buryat	Kushun	MG660630	-
17	C4a1a3	Even	Markovo	MG660633	-
18	C4a1a3	Evenki	Nelkan, Dzhigda	MG660660	C12348T
19	C4a1a3	Todzhi	Toora-Hem	MG660677	-
20	C4a1a3	Todzhi	Toora-Hem	MG660679	-
21	C4a1a3	Todzhi	Adir-Kezhig	MG660707	-
22	C4a1a3	Todzhi	Adir-Kezhig	MG660719	A2294G
23	C4a1a3a1	Tofalar	Alygdzher	MG660731	A16235G, G16390A
24	C4a1a3a1	Tofalar	Alygdzher	MG660732	A16235G, G16390A
25	C4a1a3c	Evenki	Chumikan	MG660611	-
26	C4a1a3d	Evenki	Nelkan, Dzhigda	MG660663	-
27	C4a1a4a	Evenki	Chumikan	MG660610	G12372A
28	C4a2a	Evenki	Chumikan	MG660614	T7080C
29	C4a2a1	Ket	Kellog	MG778654	A8221G, T9944C
30	C4a2a1	Evenki	Kellog	MG778683	A8221G, T9944C
31	C4a2a1	Ket	Kellog	MG778659	A8221G, T9944C
32	C4a2a1	Ket	Selivanikha	MG660619	A8221G, T9944C
33	C4a2a1	Ket	Bakhta	MG660622	-
34	C4a2a1	Ud. Buryat	Kushun	MG660626	T11147C, T16224C
35	C4a2a1	Tofalar	Nerkha	MG660648	G47A, A5984G
36	C4a2a1	Evenki	Nelkan, Dzhigda	MG660661	A5582G, T11147C
37	C4a2a1	Evenki	Val	MG660666	A5582G, T11147C
38	C4a2a1	Todzhi	Iiy	MG660699	G47A
39	C4a2a1	Tofalar	Alygdzher	MG660727	G47A, A5984G
40	C4a2a1	Tofalar	Alygdzher	MG660734	G47A, A5984G
41	C4a2a1	Tofalar	Alygdzher	MG660739	G47A, A5984G
42	C4a2a1	Tofalar	V. Gutara	MG660744	T11147C, T13879C, T16224C
43	C4a2a1	Tofalar	V. Gutara	MG660746	G47A, A5984G
44	C4a2a1a	Evenki	Nelkan, Dzhigda	MG660659	-
45	C4b	Yukaghir	Andrushkino	MG660616	-
46	C4b	Yukaghir/ Chuvan	Markovo	MG660631	T199C, C3306T, A3319G, A11928G
47	C4b	Yukaghir/ Chuvan	Markovo	MG660632	-
48	C4b	Yukaghir/ Chuvan	Markovo	MG660634	T199C, C3306T, A3319G, A11928G
49	C4b	Yukaghir/ Chuvan	Markovo	MG660635	C8410T
50	C4b	Yukaghir/ Chuvan	Markovo	MG660636	-
51	C4b	Yukaghir/ Chuvan	Markovo	MG660637	C8410T
52	C4b-16311!	Evenki	Kellog	MG778670	T16311C!
53	C4b-16311!	Tofalar	Nerkha	MG660649	C6713T, T16311C!
54	C4b-16311!	Todzhi	Toora-Hem	MG660678	A189G, C16294T, T16311C!
55	C4b-16311!	Todzhi	Toora-Hem	MG660680	A189G, C16294T, T16311C!
56	C4b-16311!	Todzhi	Iiy	MG660695	A189G, C447T, C16294T, T16311C!
57	C4b-16311!	Todzhi	Iiy	MG660701	A189G, A12425C, C16294T, T16311C!
58	C4b-16311!	Todzhi	Iiy	MG660702	T5081C, T16311C!
59	C4b-16311!	Todzhi	Adir-Kezhig	MG660709	A189G, C16294T, T16311C!
60	C4b-16311!	Todzhi	Adir-Kezhig	MG660720	C16294T, T16311C!
61	C4b-16311!	Todzhi	Adir-Kezhig	MG660723	A189G, C16294T, T16311C!
62	C4b-16311!	Tofalar	Alygdzher	MG660737	C6713T, T16311C!
63	C4b1	Evenki/Selkup	Krasnoselkup	MG778668	-
64	C4b1	Tofalar	Nerkha	MG660647	A4129G
65	C4b1	Todzhi	Toora-Hem	MG660673	A4129G
66	C4b1	Todzhi	Iiy	MG660686	A4129G
67	C4b1	Todzhi	Iiy	MG660687	A4129G
68	C4b1	Todzhi	Iiy	MG660692	A4129G
69	C4b1	Todzhi	Iiy	MG660694	G3640A, A4129G
70	C4b1	Todzhi	Iiy	MG660696	A4129G
71	C4b1	Todzhi	Iiy	MG660698	A4129G
72	C4b1	Todzhi	Iiy	MG660706	A4129G
73	C4b1	Todzhi	Adir-Kezhig	MG660721	A4129G, A11836G
74	C4b1	Tofalar	Alygdzher	MG660730	A4129G
75	C4b1	Udegey	Agzu/Ternei	MH807359	-
76	C4b1b	Evenki	Nelkan, Dzhigda	MG660662	-
77	C4b2	Koryak	Karaginskiy district	MK217927	T16223C
78	C4b2	Koryak	Karaginskiy district	MK217946	T16223C
79	C4b2a	Koryak	Karaginskiy district	MK217912	-
80	C4b2a	Koryak	Karaginskiy district	MK217913	-
81	C4b2a	Koryak	Karaginskiy district	MK217918	-
82	C4b2a	Koryak	Karaginskiy district	MK217919	-
83	C4b2a	Koryak	Karaginskiy district	MK217920	-
84	C4b2a	Koryak	Karaginskiy district	MK217921	-
85	C4b2a	Koryak	Karaginskiy district	MK217922	-
86	C4b2a	Koryak	Karaginskiy district	MK217924	-
87	C4b2a	Koryak	Karaginskiy district	MK217928	-
88	C4b2a	Koryak	Karaginskiy district	MK217929	-
89	C4b2a	Koryak	Karaginskiy district	MK217930	-

90	C4b2a	Koryak	Karaginskiy district	MK217932	-
91	C4b2a	Koryak	Karaginskiy district	MK217938	-
92	C4b2a	Koryak	Karaginskiy district	MK217939	-
93	C4b2a	Koryak	Karaginskiy district	MK217941	-
94	C4b2a	Koryak	Karaginskiy district	MK217942	-
95	C4b2a	Koryak	Karaginskiy district	MK217944	-
96	C4b2a	Koryak	Karaginskiy district	MK217947	-
97	C4b3a	Ud. Buryat	Kushun	MG660625	C8457T, C9028A(hp), G14544A
98	C4b3a	Todzhi	Toora-Hem	MG660676	-
99	C4b3a	Todzhi	Adir-Kezhig	MG660718	-
100	C4b7	Yukaghir	Andrushkino	MG660615	-
101	C4b7	Yukaghir	Andrushkino	MG660617	-
102	C4b7	Yukaghir	Andrushkino	MG660618	-
103	C5a1	Koryak	Karaginskiy district	MK217931	T4216C
104	C5a1	Todzhi	Toora-Hem	MG660674	G1211A, G14384A
105	C5a1	Todzhi	Toora-Hem	MG660681	G1211A, G14384A
106	C5a1	Todzhi	Iiy	MG660697	G1211A, G14384A
107	C5a1	Todzhi	Iiy	MG660703	G1211A, G14384A
108	C5a1	Todzhi	Adir-Kezhig	MG660716	G1211A, G14384A
109	C5a1	Todzhi	Adir-Kezhig	MG660722	G1211A, G14384A
110	C5a2b	Chukchi	Ayon	MK180567	-
111	C5a2b	Chukchi	Ayon	MK180570	-
112	C5a2b	Chukchi	Ayon	MK180579	-
113	C5a2b	Chukchi	Ayon	MK180580	A3434G
114	C5a2b	Koryak	Karaginskiy district	MK217915	-
115	C5a2b	Koryak	Karaginskiy district	MK217917	-
116	C5a2b	Koryak	Karaginskiy district	MK217933	-
117	C5a2b	Koryak	Karaginskiy district	MK217934	-
118	C5a2b	Koryak	Karaginskiy district	MK217936	-
119	C5a2b	Koryak	Karaginskiy district	MK217914	-
120	C5a2b	Koryak	Karaginskiy district	MK217923	-
121	C5a2b	Yukaghir	Nelemnoye	MG660644	-
122	C5a2b	Evenki	Nelkan, Dzhigda	MG660664	-
123	C5b1a	Tofalar	Nerkha	MG660645	A189G, 8289-8297ins9
124	C5b1a	Avam Nganasan	Ust'-Avam	MG660651	-
125	C5b1a	Avam Nganasan	Ust'-Avam	MG660653	-
126	C5b1a	Avam Nganasan	Ust'-Avam	MG660656	-
127	C5b1a	Todzhi	Toora-Hem	MG660683	8289-8297ins9
128	C5b1a	Todzhi-Tuva	Alygdzher	MG660725	8289-8297ins9
129	C5b1a	Tofalar	Alygdzher	MG660729	A189G, 8289-8297ins9
130	C5b1a	Todzhi-Tuva	Alygdzher	MG660733	8289-8297ins9
131	C5b1a	Tofalar	Alygdzher	MG660735	A189G, 8289-8297ins9
132	C5b1a	Tofalar	V. Gutara	MG660748	A189G, 8289-8297ins9
133	C5d1	Yukaghir	Cherskiy	MG660609	A14133G
134	C5d1	Evenki	Chumikan	MG660613	-
135	C5d1	Todzhi	Iiy	MG660704	A9448G
136	D3	Yukaghir	Yanranay	MK180574	-
137	D3	Avam Nganasan	Ust'-Avam	MG660652	C4102A, A10042G
138	D3	Avam Nganasan	Ust'-Avam	MG660654	-
139	D3	Avam Nganasan	Ust'-Avam	MG660655	-
140	D3	Vadei Nganasan	Factoriya Novaya, Khatanga	MG660657	-
141	D3	Vadei Nganasan	Factoriya Novaya, Khatanga	MG660658	-
142	D4b1a2a1	Koryak	Karaginskiy district	MK217943	T11383C, A14122C, T16093C, C16519T
143	D4b1a2a1	Chukchi	Ayon	MK180572	T11383C, G11914A!, A14122C, T16093C, C16519T
144	D4b1a2a1	Chukchi	Ayon	MK180566	T11383C, A14122C, C15370T, T16093C, C16519T
145	D4b1a2a1	Chukchi	Yanranay	MK180577	T11383C, G11914A!, A14122C, T16093C, C16519T
146	D4b1a2a2	Tuvan	Bai-Tal	MG660671	G14207A, C16519T
147	D4b1a2a2	Todzhi	Iiy	MG660690	-
148	D4b1a2a2	Todzhi	Iiy	MG660691	-
149	D4j4	Evenki	Nelkan, Dzhigda	MG660665	T14470C
150	D4j4a	Evenki/Ulta	Val	MG660667	C16148T
151	D4j8	Ud. Buryat	Kushun	MG660629	-
152	D4p	Todzhi	Iiy	MG660705	-
153	D4p	Todzhi	Adir-Kezhig	MG660710	-
154	D5a2a1-16172!	Todzhi	Adir-Kezhig	MG660711	T152C!, A16183C
155	F1b1b	Ket	Krasnoselkup	MG778671	T10227C, C16179T, A16183C
156	F1b1b	Todzhi	Toora-Hem	MG660685	A16183C, C16286T
157	F1b1b	Todzhi	Iiy	MG660689	A16183C, C16286T
158	F1b1b	Todzhi	Iiy	MG660693	G203A, C3204T, T10227C, C16179T, A16183C
159	F1b1b	Todzhi	Iiy	MG660700	G203A, C3204T, T10227C, C16179T, A16183C
160	F1b1b	Todzhi	Adir-Kezhig	MG660708	G203A, C3204T, T10227C, C16179T, A16183C
161	F1b1b	Todzhi	Adir-Kezhig	MG660713	G203A, C3204T, T10227C, C16179T, A16183C
162	F1b1b	Todzhi	Adir-Kezhig	MG660714	G203A, C3204T, T10227C, C16179T, A16183C
163	F1b1b	Todzhi	Adir-Kezhig	MG660717	G203A, C3204T, T10227C, C16179T, A16183C
164	F1b1b	Tofalar	Alygdzher	MG660728	A16183C, C16286T
165	F1b1e	Ud. Buryat	Alygdzher	MG660736	A515G, A16183C
166	G1a1	Todzhi	Toora-Hem	MG660682	G4113A, A11908G, A14569G!, C15745A, C16260T
167	G2b2	Todzhi	Adir-Kezhig	MG660715	T152C!, T1709A, T5641C, A6647T, T11984C, A14569G!, T16093C
168	H101*	Ket	Maduika	MG778680	T152C!, T9230C, A12290G, A15401C
169	H1ae3*	Ket	Kellog	MG778661	T1189C, G8723A
170	H1b2	Ket	V.Baikha	MG778677	T6293C, G16129A!, A16183C
171	J1c2m	Ket	Sov. Rechka-Pakulikha	MG778682	A3918G
172	M8a1	Udegey	Bogorodskoye	MG660741	A234G, A3744G
173	M8a1	Udegey	Agzu/Ternei	MH807364	A234G, A3744G
174	M8a2b	Nanai	Bogorodskoye	MG660742	G12192A, G15106A
175	M8a3	Ud. Buryat	Kushun	MG660627	T146C!, T152C!, G5237A, T13488C, A16293G
176	M8a3	Tofalar	Nerkha	MG660646	T146C!, T152C!, G5237A, T13488C, A16293G
177	M8a3	Tofalar	Nerkha	MG660650	T146C!, T152C!, G5237A, T13488C, A16293G
178	M8a3	Tofalar	V. Gutara	MG660745	T146C!, T152C!, G5237A, T13488C, A16293G
179	M8a3	Tofalar	V. Gutara	MG660747	T146C!, T152C!, G5237A, T13488C, A16293G
180	M8a3	Tofalar	V. Gutara	MG660749	T146C!, T152C!, G5237A, T13488C, A16293G
181	M8a3	Tofalar	V. Gutara	MG660750	T146C!, T152C!, G5237A, T13488C, A16293G

182	M8a3a	Russian	Turukhansk	MG778679	T146C!, T11260C, G13590A, A15684G, T16189C!, T16311C!
183	N2a	Ket	Kellog	MG778664	T152C!, T1633C, T11722C, G12192A
184	U3b	Todzhi	Toora-Hem	MG660684	G2652A, T13768C
185	U3b	Todzhi	Adir-Kezhig	MG660712	G2652A, T10885C, T13768C
186	U4a1	Ket	Kellog	MG778660	T72C, A12950G, T14635C
187	U4a1	Ket	Maduika	MG778665	T72C, A12950G, T14635C
188	U4a1	Ket	Farkovo	MG778655	T72C, A12950G, T14635C
189	U4a1	Ket	Kellog	MG778667	T72C, A12950G, T14635C
190	U4a1	Ket	Kellog	MG778657	T72C, A12950G, T14635C
191	U4a1	Ket	Pakulikha	MG778678	T72C, A12950G, T14635C
192	U4a1	Ket	Farkovo	MG778681	T72C, A12950G, T14635C
193	U4a1	Ket	Surgutikha	MG778658	T72C, A12950G, T14635C
194	U4d2	Ket	Surgutikha	MG778674	C16111T, G16390A
195	U4d2	Ket	Surgutikha	MG778675	C16111T, G16390A
196	U4d2	Ket	Vorogovo	MG778676	A8596G, A12234G, C16189T!!
197	U4d2	Mansi	Shaim	MG660638	G10320A
198	U4d2	Mansi	Urai	MG660642	G10320A
199	U5a1d2	Ket	Kellog	MG778669	T146C!, T4823C
200	U5a1d2	Ket	Baikha	MG660620	T146C!, T4823C
201	U5a1d2	Ket	Kellog	MG660621	T146C!, T4823C
202	U5a1h*	Mansi	Aneyeva	MG660643	C150T, C3192T, G3591A, A6895G, G12618A, A13966G, G16129A!, T16192C!, C16239T
203	U5a2a1b	Mansi	Verkhoturye	MG660639	A9115G
204	U5a2b	Mansi	Shaim	MG660640	C6713T, T16311C!
205	U5b2b	Ud. Buryat	Kushun	MG660623	T8227C, T12696C, T15900C(hp), T16093C, C16223T!, C16234T(hp)
206	U5b2b	Tuvan	Bai-Tal	MG660668	T7964C, T8077C, T9185G, G13968T, G14040A, T16189C!, C16260T
207	U5b2b	Tuvan	Bai-Tal	MG660669	T7964C, T8077C, T9185G, G13968T, G14040A, A16183C, T16189C!, C16260T
208	Y1a1	Ket	Yelogui	MG778663	T3394C
209	Y1a1	Ket	Yelogui	MG778656	T3394C
210	Y1a1	Ket	Kellog	MG778672	T3394C
211	Y1a1	Ket	Kellog	MG778673	-
212	Z1	Tofalar	Alygdzher	MG660740	G207A, G251A, A3434G, T6425C, G9948A, T14180C, C16360T
213	Z1a	Koryak	Karaginskiy district	MK217935	-
214	Z1a	Koryak	Karaginskiy district	MK217945	-
215	Z1a1a	Ket	Kangatovo	MG778666	-
216	Z1a2a	Koryak	Karaginskiy district	MK217916	-
217	Z1a2a	Koryak	Karaginskiy district	MK217937	-
218	Z1a2a	Koryak	Karaginskiy district	MK217940	-
