# Supplementary material for: Mitochondrial genome diversity on the Central Siberian Plateau with particular reference to the prehistory of northernmost Eurasia
Source: PLoS One. 2021 Jan 28;16(1):e0244228. doi: 10.1371/journal.pone.0244228 (PMC7842996; doi:10.1371/journal.pone.0244228)
Supplement: S1 File — (ZIP) [file pone.0244228.s001.zip › S2_Table.pdf]

Supplemental Table S2. List of ancient mtDNA samples used in this study. In bold are newly generated.

#	Sample ID	mtDNA haplotype	Date	Culture/Arch.Period	Location/Cite	Country	Latitude	Longitude	Publication
1	DA344	A+152+16362	4885 BP	LN	Ust'-Ida	Russia	53,188889	103,368056	Damgaard et al. 2018
2	DA357	A+152+16362	6713 BP	EN	Lokomotiv, Cis-Baikal	Russia	52,286944	104,249167	Damgaard et al. 2018
3	RISE674	A+152+16362	4061 BP (2281-1976 BC cal)	Okunevo, EMBA	Verkhni Askiz	Russia	53,156486	90,207811	Damgaard et al. 2018
4	RISE680	A+152+16362	4329 BP	Okunevo, EMBA	Uybat V	Russia	53,708561	90,359808	Damgaard et al. 2018
5	RISE497	A+152+16362	1400-900 BCE	Karasuk	Arban 1	Russia	52.954	90.187	Allentoft et.al. 2015
6	irk040	A+152+16362	N/A	Neolithic Cis-Baikal	Gorodische N 1 (Angara river, Irkutsk Oblast)	Russia	53.220694	103.39475	Kilinç et al. 2018
7	irk025	A+152+16362	cal BC 2475 to 2335, cal BC 2325 to 2300	Bronze Age Cis-Baikal	Sukhaja Pad' Buret' site, burial 2	Russia	52.983756	103.520719	Kilinç et al. 2018
8	brn001	A+152+16362	cal BC 5490 to 5465, cal BC 5400 to 5385	Mesolithic Trans-Baikal	Izvestkovaja-1 site, burial 2 (Kuenga river, Sretensky District)	Russia	52.230833	116.993333	Kilinç et al. 2018
9	irk030	A10	N/A	Neolithic Cis-Baikal	Korkino, burial 1 (Upper Lena river)	Russia	54.373847	105.209969	Kilinç et al. 2018
10	N4a1	A12a	cal BC 2830 to 2820, cal BC 2625 to 2475	Late Neolithic Yakutia	Kyordyughen 2 (Central Yakutia)	Russia	62.066667	132.333611	Kilinç et al. 2018
11	N4b2	A12a	cal BC 2475 to 2295	Late Neolithic Yakutia	Kyordyughen 1, burial 1 (Central Yakutia)	Russia	62.066667	132.333333	Kilinç et al. 2018
12	I0562	A16	400-300 BCE	Kazakhstan_Berel_IA	Berel	Kazakhstan	49.3356	86.350367	Unterlander et al. 2017
13	RISE664	A8a1	4272 BP (2459-2206 BC cal)	Okunevo, EMBA	Okunev Ulus	Russia	53,547806	91,02565	Damgaard et al. 2018
14	RISE677	A8a1	4419 BP (2831-2233 BC cal)	Okunevo, EMBA	Uybat III	Russia	53,708561	90,359808	Damgaard et al. 2018
15	RISE681	A8a1	4329 BP	Okunevo, EMBA	Uybat V	Russia	53,708561	90,359808	Damgaard et al. 2018
16	RISE515	A8a2	4197 BP (2340-2145 BC cal)	Okunevo, EMBA	Verkhni Askiz	Russia	53,156486	90,207811	Damgaard et al. 2018
17	RISE667	A8a2	4078.5 BP	Okunevo, EMBA	Verkhni Askiz	Russia	53,156486	90,207811	Damgaard et al. 2018
18	RISE670	A8a2	4088 BP (2141-1885 BC cal)	Okunevo, EMBA	Verkhni Askiz	Russia	53,156486	90,207811	Damgaard et al. 2018
19	RISE673	A8a2	4078.5 BP	Okunevo, EMBA	Verkhni Askiz	Russia	53,156486	90,207811	Damgaard et al. 2018
20	RISE515	A8a2	3810 BP (2340-2145 BC)	Okunevo	Verkhni Askiz	Russia	53.153	90.194	Allentoft et.al. 2015
21	mak026	C4	cal BC 2895 to 2860, cal BC 2805 to 2755, cal BC 2720 to 2705	Bronze Age Cis-Baikal	Makrushynskij burial site, burial 26 (Upper Lena river)	Russia	53.876672	106.267394	Kilinç et al. 2018
22	irk057	C4	cal BC 2550 to 2535, cal BC 2490 to 2395, cal BC 2385 to 2345	Bronze Age Cis-Baikal	Podostrozhnoe N 3 (Angara river, Irkutsk Oblast)	Russia	53.220694	103.39475	Kilinç et al. 2018
**23**	**I1000**	**C4**	**2871-2497 calBCE (4100±40 BP, Poz-83436)**	**Glazkovskaya**	**Obkhoy, Kachugskiy district, Irkutsk region**	**Russia**	**54.0186686**	**105.4747222**	
24	DA247	C4	6856 BP	EN	Shamanka II	Russia	51,698333	103,703056	Damgaard et al. 2018
25	DA248	C4	6815 BP	EN	Shamanka II	Russia	51,698333	103,703056	Damgaard et al. 2018
26	DA249	C4	7005 BP	EN	Shamanka II	Russia	51,698333	103,703056	Damgaard et al. 2018
**27**	**I2072**	**C4+152**	**3959-3715 calBCE (5050±40 BP, Poz-83497)**	**Solentsy5_N**	**Solontcy 5, Foothhills of the Altai**	**Russia**	**52.4833**	**86.2167**	
28	RISE602	C4+152	9/700 BC - AD 500/1000	Iron Age	Sary-Bel	Russia	50.615	84.459	Allentoft et.al. 2015
29	I0563	C4a1a	400-300 BCE	Kazakhstan_Berel_IA	Firsovo-XI, Ob bank, Altai province	Kazakhstan	49.3356	86.350367	Unterlander et al. 2017
30	DA337	C4a1a3	3871 BP	EBA	Shamanka II	Russia	51,698333	103,703056	Damgaard et al. 2018
31	DA356	C4a1a3	3854 BP	EBA	Ust'-Ida	Russia	53,188889	103,368056	Damgaard et al. 2018
32	DA361	C4a1a3	3854 BP	EBA	Ust'-Ida	Russia	53,188889	103,368056	Damgaard et al. 2018
33	irk033	C4a1a3	cal BC 2920 to 2880	Bronze Age Cis-Baikal	Chastaja Padi (Angara river, Irkutsk Oblast)	Russia	52.989242	103.450869	Kilinç et al. 2018
34	irk008	C4a1a3	cal BC 5615 to 5485	Mesolithic Trans-Baikal	Izvestkovaja-1 site, burial 1 (Kuenga river, Sretensky District)	Russia	54.002744	105.710294	Kilinç et al. 2018
35	irk076	C4a2a1	cal BC 2275 to 2250, cal BC 2225 to 2220, cal BC 2210 to 2120 and cal BC 2090 to 2040	Bronze Age Cis-Baikal	Shamanka 2, burial 3 (South Baikal)	Russia	51.694478	103.70475	Kilinç et al. 2018
36	DA334	C4a2a1	3764 BP	EBA	Shamanka II	Russia	51,698333	103,703056	Damgaard et al. 2018
37	DA336	C4a2a1	3817.5 BP	EBA	Shamanka II	Russia	51,698333	103,703056	Damgaard et al. 2018
38	DA338	C4a2a1	3817.5 BP	EBA	Shamanka II	Russia	51,698333	103,703056	Damgaard et al. 2018
39	yak021	C4b+16311!	cal BC 1385 to 1340, cal BC 1315 to 1195, cal BC 1140 to 1130	Late Neolithic Yakutia	Pomazkino site, burial 2 (Kolyma river)	Russia	67.916667	156.5	Kilinç et al. 2018
40	kra011	C4b1	cal BC 2295 to 2140	Neolithic -Bronze? Krasnoyarsk Krai	Nefteprovod-2 site, burial 1 (Krasnoyarsk Krai)	Russia	56.194736	95.819539	Kilinç et al. 2018
41	yak022	C4b1	cal BC 1940 to 1765	Late Neolithic Yakutia	Kamenka 2 burial, individual 1 (Kolyma river)	Russia	66.8	152.633333	Kilinç et al. 2018
42	yak023	C4b1	cal BC 1880 to 1690	Late Neolithic Yakutia	Kamenka 2 burial, individual 2 (Kolyma river)	Russia	66.8	152.633333	Kilinç et al. 2018
43	yak024	C4b1	in the same burial with yak022, yak023	Late Neolithic Yakutia	Kamenka 2 burial, individual 3 (Kolyma river)	Russia	66.8	152.633333	Kilinç et al. 2018
44	N3a	C4b3	cal BC 790 to 730, cal BC 690 to 660, cal BC 650 to 540	Iron Age Yakutia	Dyupsya burial (Central Yakutia)	Russia	63.025	130.73	Kilinç et al. 2018
45	irk078	C5	cal BC 1260 to 1050	Bronze Age Trans-Baikal	Okoshki 1, burial 23 (Zabaykalsky Krai)	Russia	50.317222	118.275361	Kilinç et al. 2018
46	irk00x	C5+16093	cal BC 6500 to 6435	Mesolithic Trans-Baikal	Dzhylinda site (Chitinsuyu area)	Russia	55.668922	115.872081	Kilinç et al. 2018
47	RISE684	C5c	4239 BP (2464-2141 BC cal)	EBA	Uybat V	Russia	53,708561	90,359808	Damgaard et al. 2018
48	RISE685	C5c	4329 BP	EBA	Uybat V	Russia	53,708561	90,359808	Damgaard et al. 2018
49	RISE718	C5c	4436 BP (2573-2348 BC cal)	EBA	Syda 5, Tumen	Russia	54,371767	91,506856	Damgaard et al. 2018
50	RISE719	C5c	4450 BP	EBA	Syda 5, Tumen	Russia	54,371767	91,506856	Damgaard et al. 2018
51	irk022	D4b1c	cal BC 2455 to 2200	Bronze Age Cis-Baikal	Ust'-Dolgoe site, burial 3	Russia	52.994322	103.420928	Kilinç et al. 2018
52	N5a	D4b1c	cal BC 4340 to 4235	Middle Neolithic Yakutia	Onnyos burial (Amga river)	Russia	60.458333	131.091667	Kilinç et al. 2018
53	DA358	F1b	4169 BP	EBA	Kurma XI	Russia	53,179167	106,962778	Damgaard et al. 2018
54	DA360	F1b	4158 BP	EBA	Kurma XI	Russia	53,179167	106,962778	Damgaard et al. 2018
55	DA253	F1b1+152	6329 BP	EN	Shamanka II	Russia	51,698333	103,703056	Damgaard et al. 2018
56	DA335	F1b1b	3818 BP	EBA	Shamanka II	Russia	51,698333	103,703056	Damgaard et al. 2018
57	irk036	F1b1b(2)	cal BC 2885 to 2835, cal BC 2815 to 2665	Bronze Age Cis-Baikal	Glazkovo (Angara river, Irkutsk Oblast)	Russia	52.286378	104.260106	Kilinç et al. 2018
58	RISE553	F1b1b2	2731 BP (926-815 BC)	LBA	Afontova Gora, Krasnoyarsk	Russia	56.016	92.866	Allentoft et.al. 2015
59	irk068	F1b1b3	N/A	Neolithic Cis-Baikal	Shishkino N 1 (Upper Lena river)	Russia	54.006611	105.681531	Kilinç et al. 2018
60	RISE554	F1b1b3	2782 BP (1005-844 BC)	LBA	Afontova Gora, Krasnoyarsk	Russia	56.016	92.866	Allentoft et.al. 2015
61	I0211	U4a	6773-5886 BCE	Karelia_HG	Yuzhnyy Oleni Ostrov, Karelia	Russia	61.65	35.65	Mathieson et.al. 2015
**62**	**I0992**	**U4a(3)**	**5002-4730 calBCE (5990±50 BP, Poz-83428)**	**Novosibirsk_N**	**Korchugan-1, Novosibirsk region**	**Russia**	**56.4666647**	**76.3**	
**63**	**I2074**	**U4a(3)**	**5602-5376 calBCE (6520±40 BP, Poz-83514)**	**Kemerovo_N**	**Vas'kovo-4, burial 1, Intermountain basin between spurs of Altai and Sayan Mountains**	**Russia**	**55.0537**	**85.0966**	
64	I0231	U4a1	2921-2762 calBCE (4260±30 BP, Beta-392487)	Yamnaya_Samara	Ekaterinovka, Southern Steppe, Samara	Russia	52.42	48.24	Mathieson et.al. 2015
65	SBj	U4a1	8963-8579 calBP	Mesoltihic Scandinavian Hunter-gatherer	Stora Bjers	Sweden	57.8167	18.53	Gunther et.al. 2018
66	SF12	U4a1	9033–8757 calBP	Mesoltihic Scandinavian Hunter-gatherer	Stora Förvar	Sweden	57.2853	17.9706	Gunther et.al. 2018
67	I0434	U4d	5200-4000 BCE	Samara_Eneolithic	Khvalynsk II, Volga River, Samara	Russia	52.22	48.1	Mathieson et.al. 2015
68	RISE500	U4d1	1700-1500 BC	Andronovo	Kytmanovo	Russia	53.456	85.447	Allentoft et.al. 2015
69	I0124	U5a1d	5657-5541 calBCE (6680±30 BP, Beta-392490)	Samara_HG	Lebyazhinka IV, Sok River, Samara	Russia	53.4	50.4	Mathieson et.al. 2015
70	Hum2	U5a1d	9452-9275 calBP	Mesoltihic Scandinavian Hunter-gatherer	Hummervikholmen, Søgne archipelago, Southern Norway	Norway	58.064	7.7438	Gunther et.al. 2018
71	Steigen	U5a1d	5950-5764 calBP	Mesoltihic Scandinavian Hunter-gatherer	Måløya, Steigen, Northern Norway	Norway	67.81	14.6818	Gunther et.al. 2018
72	RISE502	U5a1d	3140 BP (1496-1306 BC)	Karasuk	Bystrovka	Russia	51.909	88.574	Allentoft et.al. 2015
73	RISE240	U5a1d1	4160 BP (2880-2632 BC)	Yamnaya	Sukhaya Termista I	Russia	46.58	43.678	Allentoft et.al. 2015
**74**	**I2068**	**U5a1d2**	**420-565 calCE (1560±30 BP, Poz-83507)**	**Siberia_IA**	**Tepsei III, kurgan 2, Minusinskaya intermauntain basin, Sayan Mountain**	**Russia**	**53.9634**	**91.5587**	
75	RISE546	U5a1d2b	3000-2400 BC	Yamnaya	Temrta IV	Russia	46.539	43.699	Mathieson et.al. 2015
**76**	**I0991**	**Z1**	**5206-4805 calBCE (6060±50 BP, Poz-83427)**	**Novosibirsk_N**	**Korchugan-1, Novosibirsk region**	**Russia**	**56.4666647**	**76.3**	
**77**	**I0998**	**Z1a1**	**2835-2472 calBCE (4040±35 BP, Poz-83426)**	**Serovskaya**	**Khuzhir, Lake Baikal, Olkhon Island, Irkutsk region**	**Russia**	**53.193333**	**107.343889**	
