# Supplementary figures and images for: Mitochondrial genome diversity on the Central Siberian Plateau with particular reference to the prehistory of northernmost Eurasia
Source: PLoS One. 2021 Jan 28;16(1):e0244228. doi: 10.1371/journal.pone.0244228 (PMC7842996; doi:10.1371/journal.pone.0244228)

Figure S1.

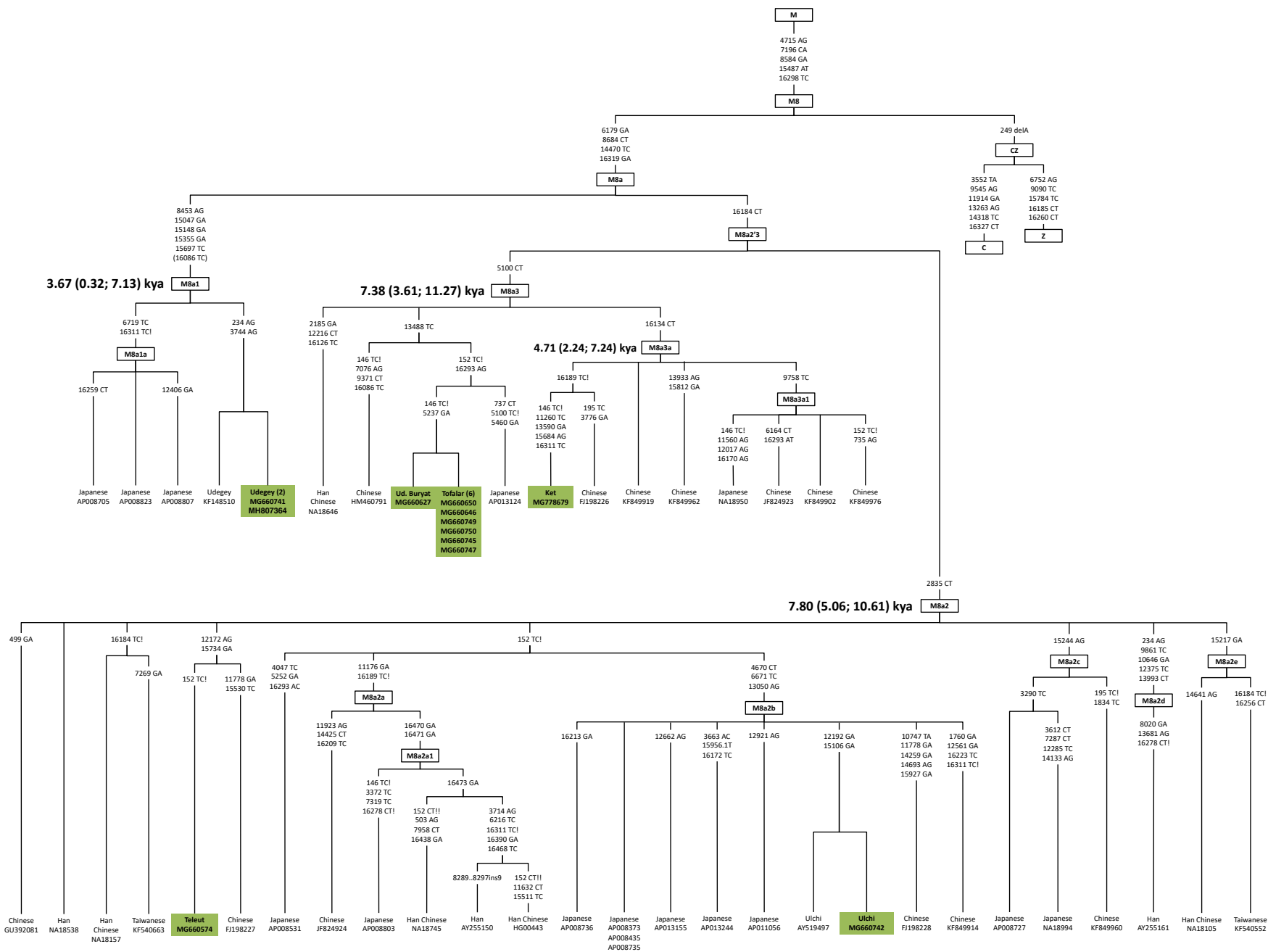

Supplement: S1 File — (ZIP) [file pone.0244228.s001.zip › S1_Fig.pdf]

**Figure S10.**

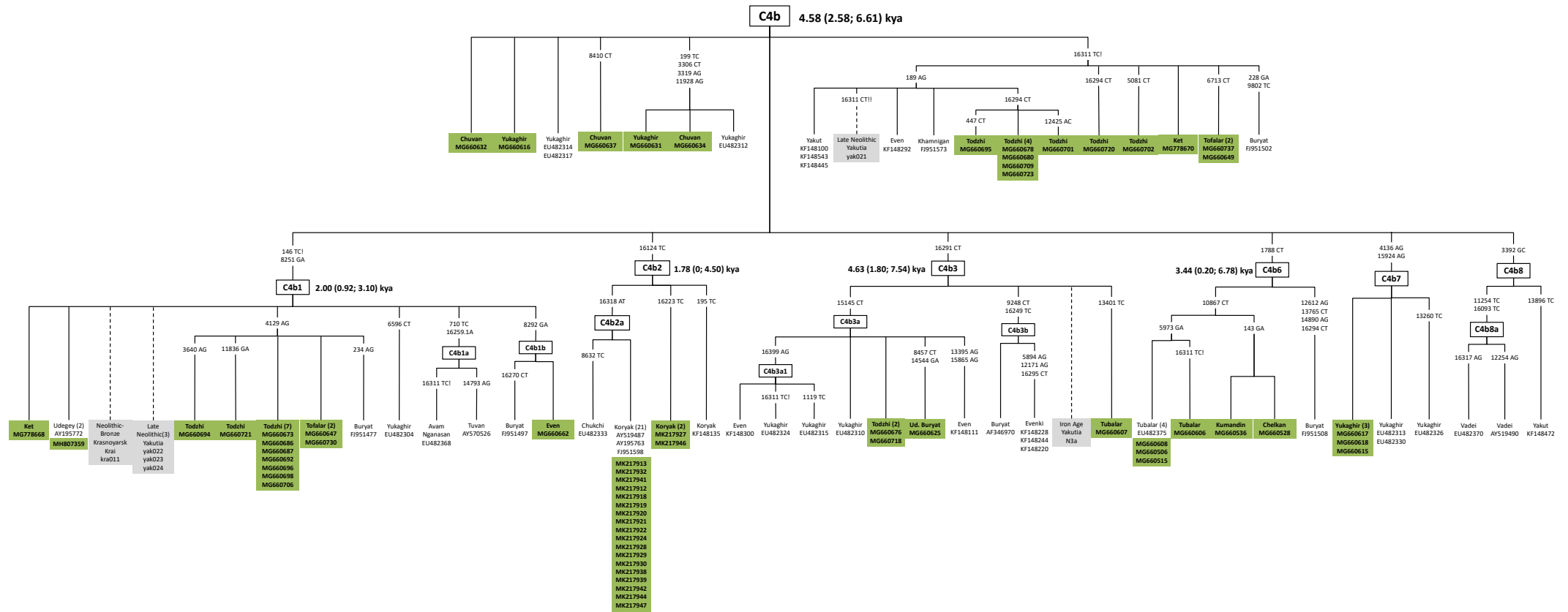

Supplement: S1 File — (ZIP) [file pone.0244228.s001.zip › S10_Fig.pdf]

Figure S11.

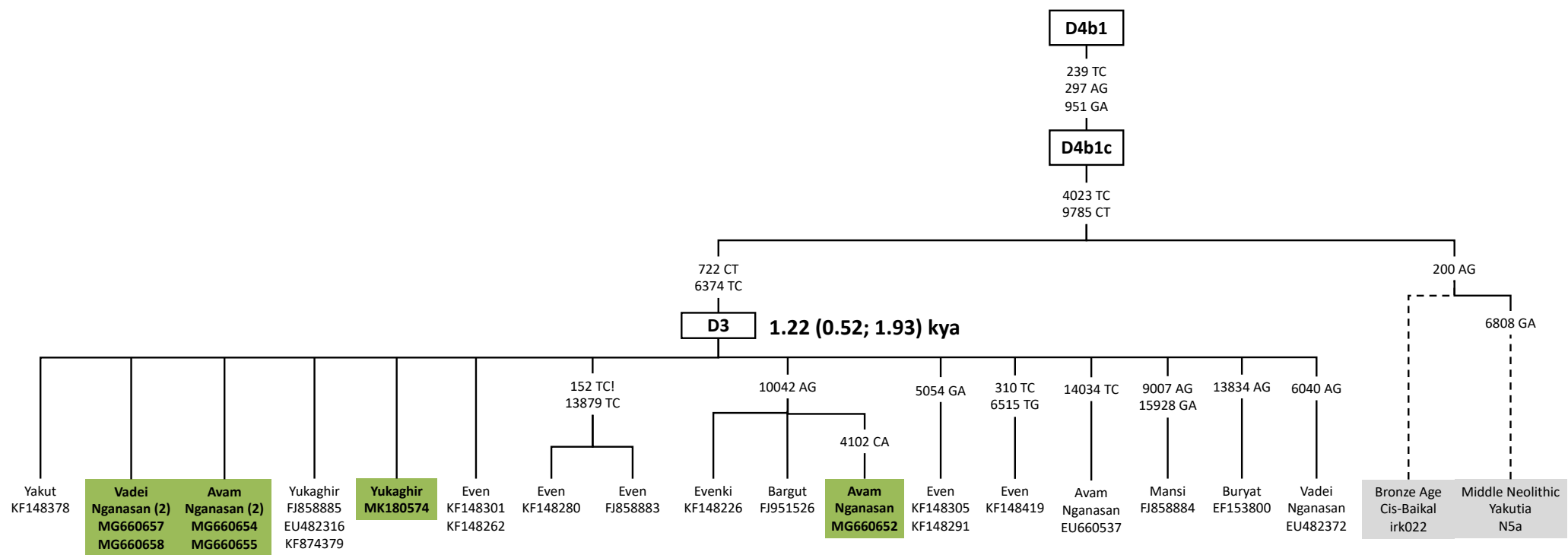

Supplement: S1 File — (ZIP) [file pone.0244228.s001.zip › S11_Fig.pdf]

**Figure S2.**

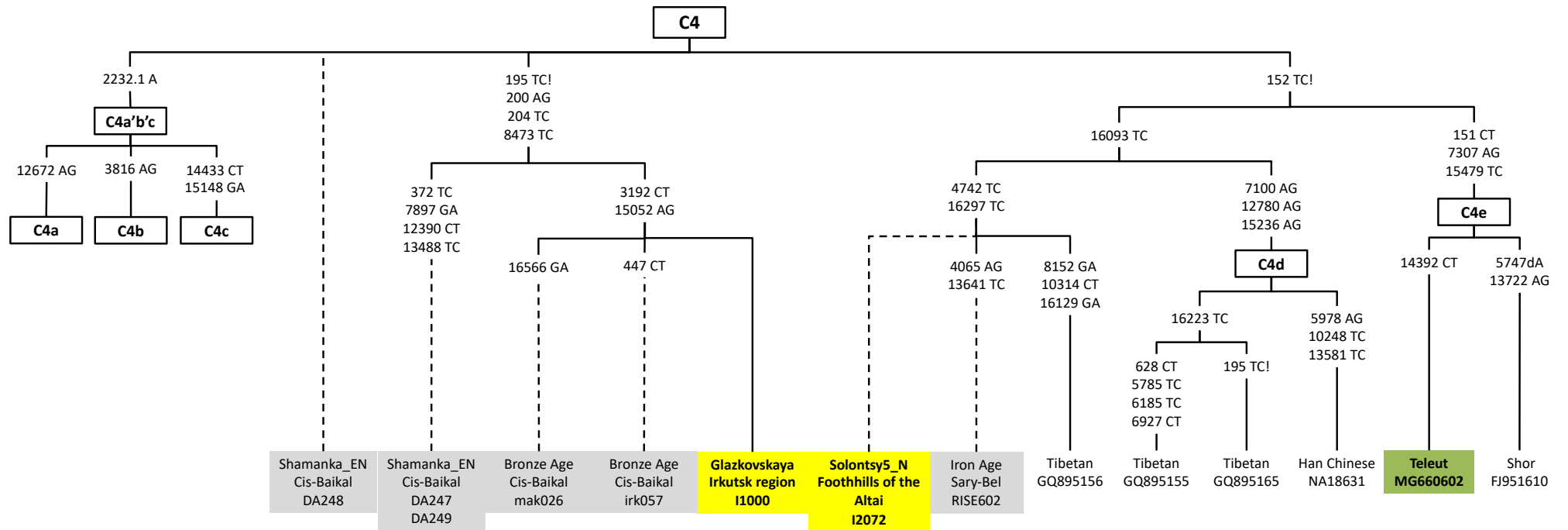

Supplement: S1 File — (ZIP) [file pone.0244228.s001.zip › S2_Fig.pdf]

Figure S3.

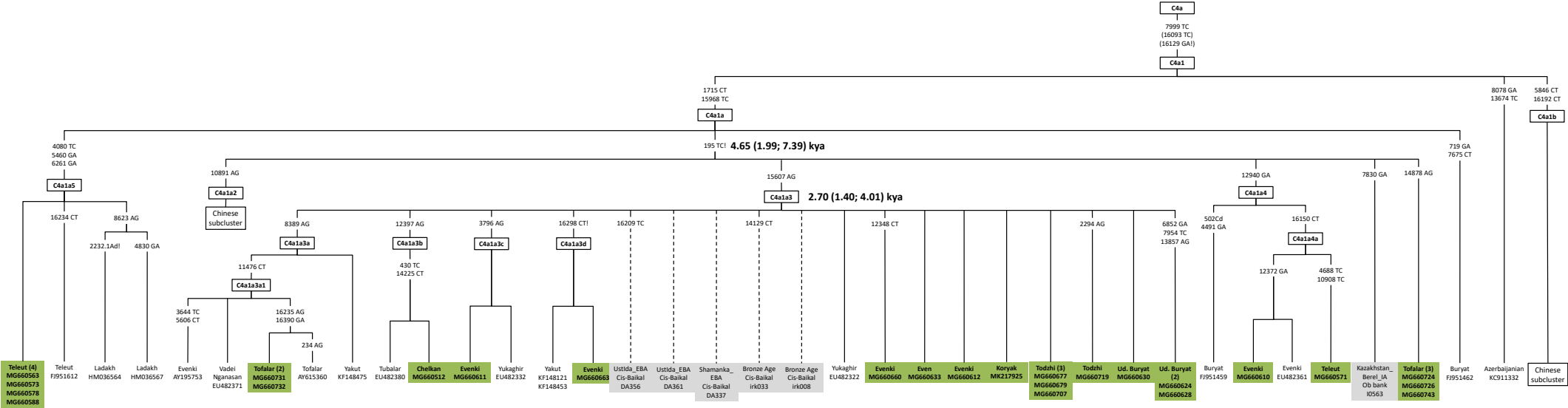

Supplement: S1 File — (ZIP) [file pone.0244228.s001.zip › S3_Fig.pdf]

Figure S4.

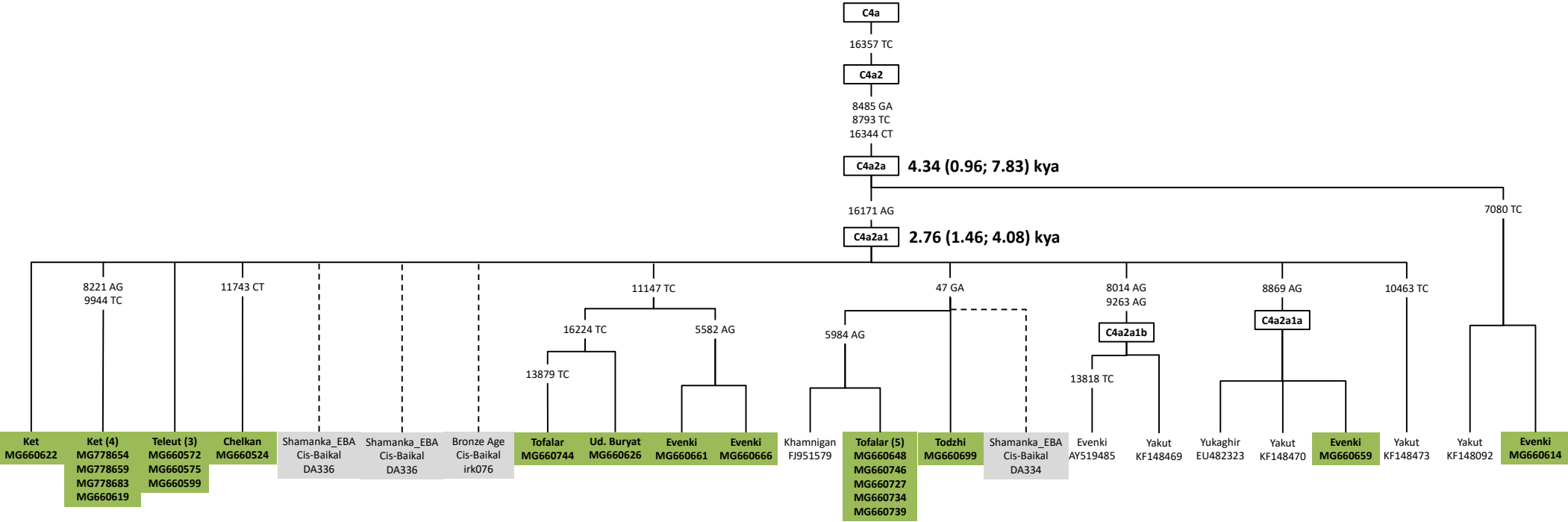

Supplement: S1 File — (ZIP) [file pone.0244228.s001.zip › S4_Fig.pdf]

Figure S5.

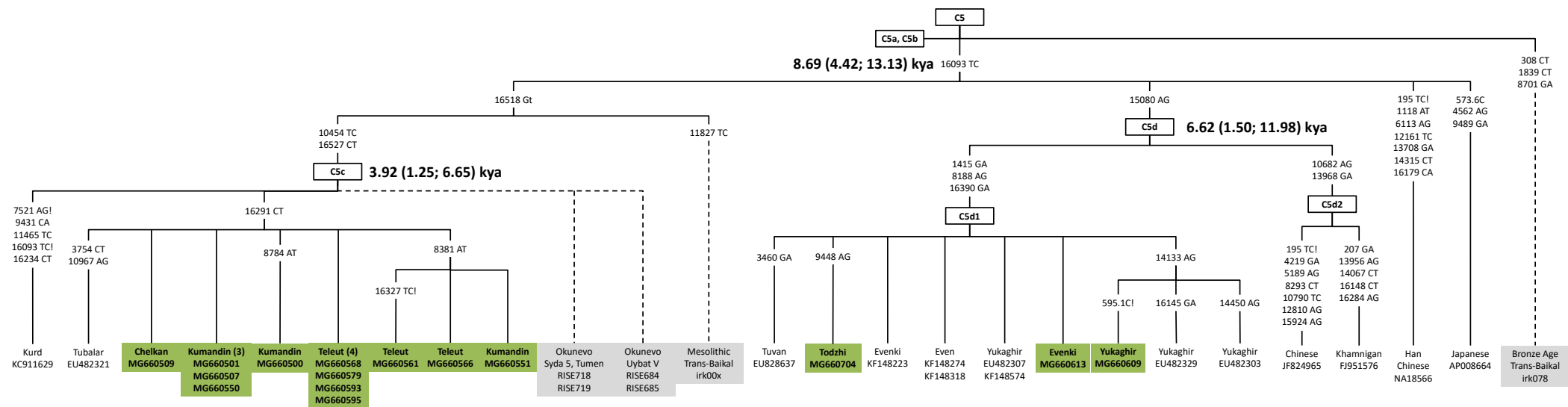

Supplement: S1 File — (ZIP) [file pone.0244228.s001.zip › S5_Fig.pdf]

Figure S6.

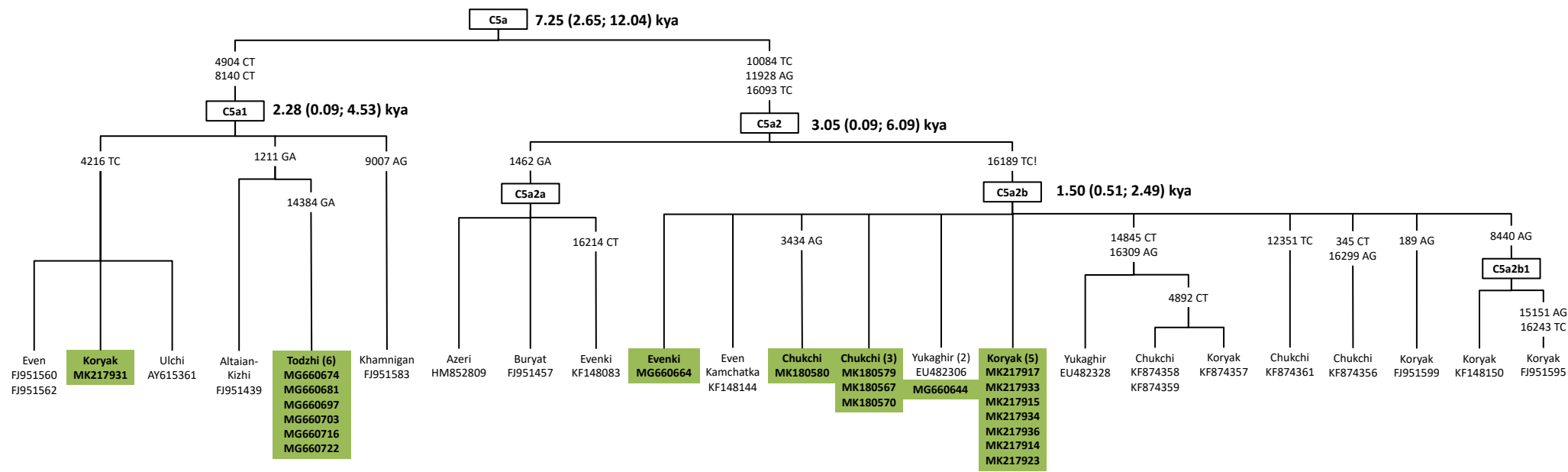

Supplement: S1 File — (ZIP) [file pone.0244228.s001.zip › S6_Fig.pdf]

Figure S7.

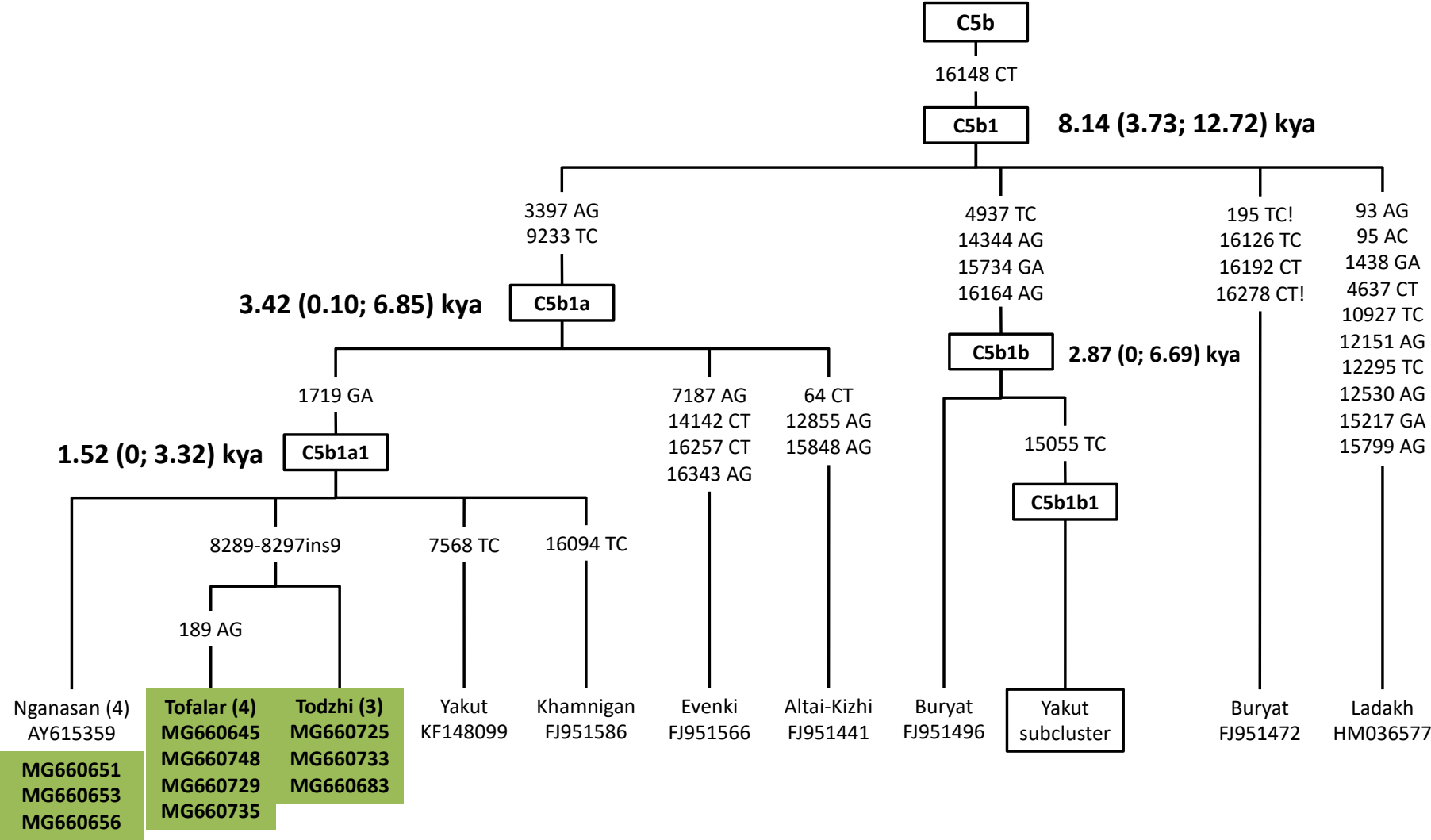

Supplement: S1 File — (ZIP) [file pone.0244228.s001.zip › S7_Fig.pdf]

**Figure S8.**

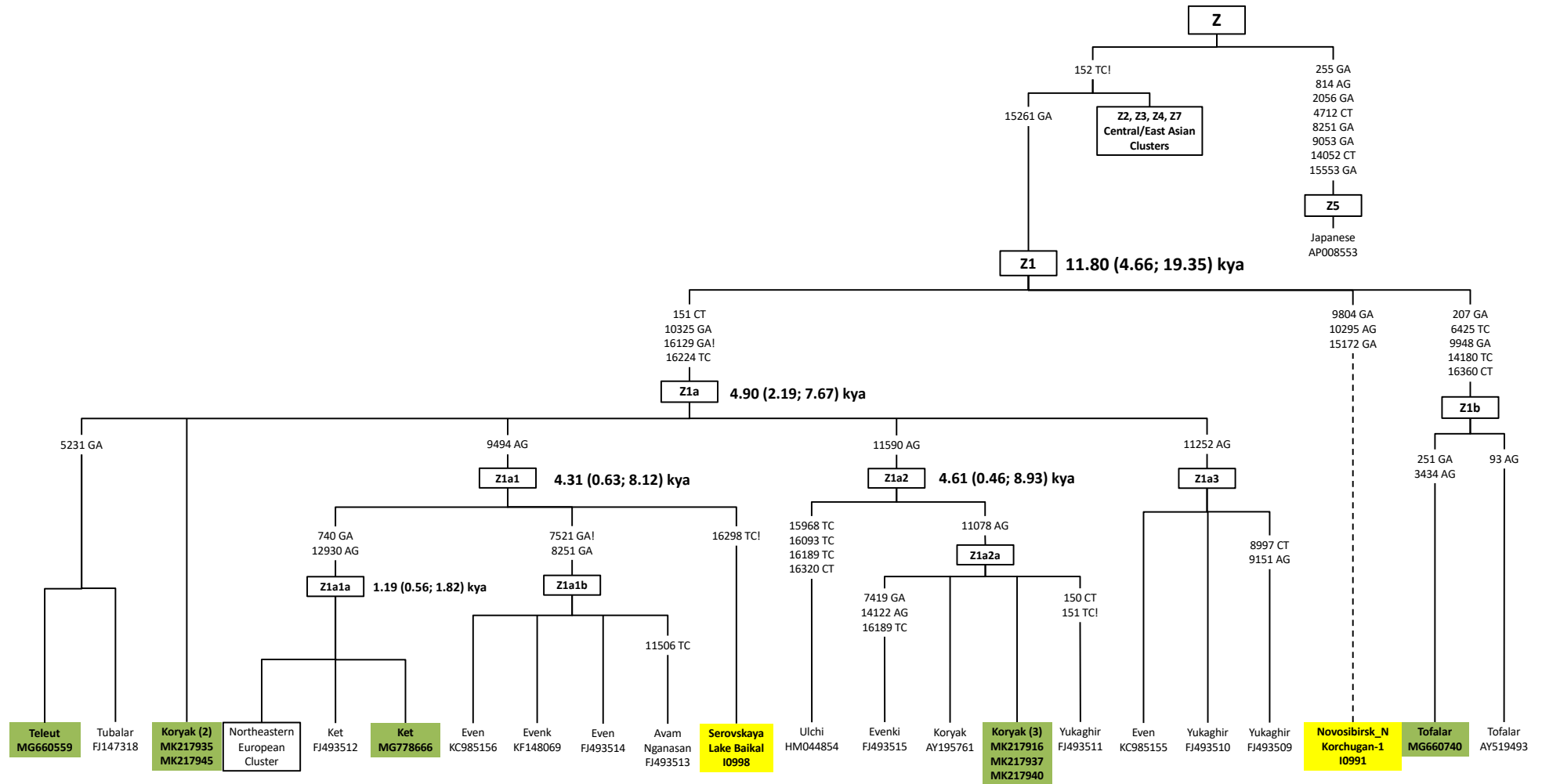

Supplement: S1 File — (ZIP) [file pone.0244228.s001.zip › S8_Fig.pdf]

Figure S9.

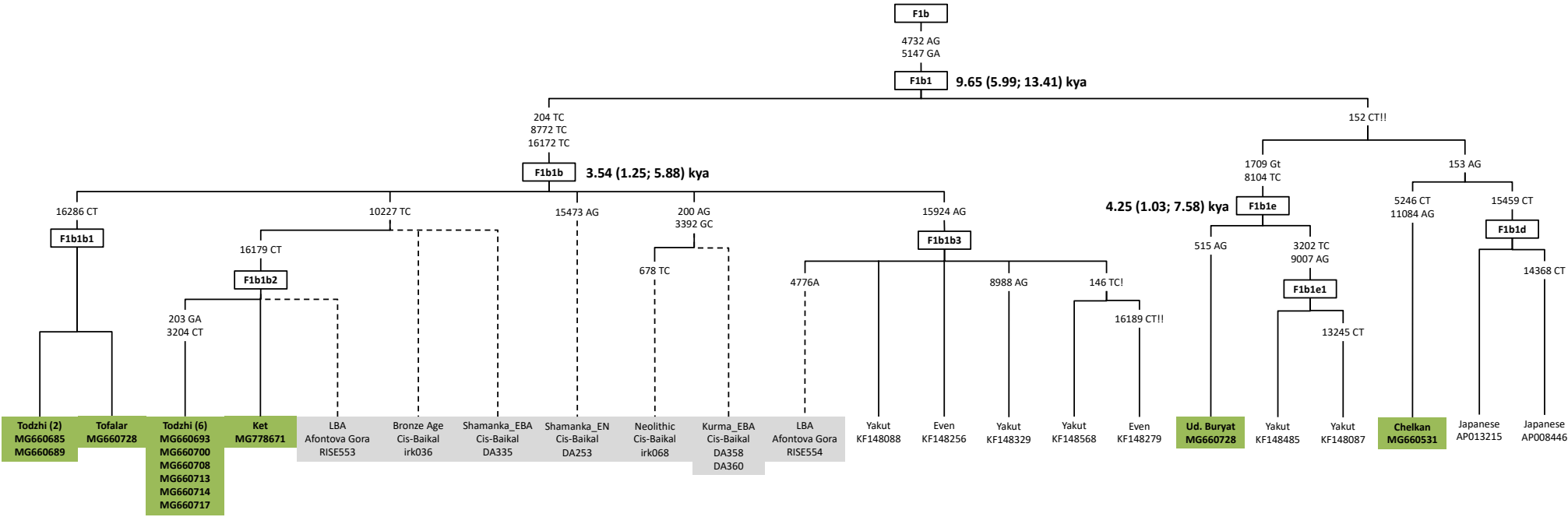

Supplement: S1 File — (ZIP) [file pone.0244228.s001.zip › S9_Fig.pdf]
